# Atractylenolide I inhibits angiogenesis and reverses sunitinib resistance in clear cell renal cell carcinoma through ATP6V0D2-mediated autophagic degradation of EPAS1/HIF2α

**DOI:** 10.1080/15548627.2024.2421699

**Published:** 2024-10-30

**Authors:** Qinyu Li, Kai Zeng, Qian Chen, Chenglin Han, Xi Wang, Beining Li, Jianping Miao, Bolong Zheng, Jihong Liu, Xianglin Yuan, Bo Liu

**Affiliations:** a Department of Urology, Tongji Hospital, Tongji Medical College, Huazhong University of Science and Technology, Wuhan, Hubei, China; b Department of Urology, The First Affiliated Hospital of Shihezi University, Shihezi, Xinjiang, China; c Hepatic Surgery Center, Tongji Hospital, Tongji Medical College, Huazhong University of Science and Technology, Wuhan, Hubei, China; d Department of Hepatobiliary Surgery, The First Affiliated Hospital of Shihezi University, Shihezi, Xinjiang, China; e Department of Oncology, Tongji Hospital, Tongji Medical College, Huazhong University of Science and Technology, Wuhan, Hubei, China; f Department of Geriatrics, Tongji Hospital, Tongji Medical College, Huazhong University of Science and Technology, Wuhan, Hubei, China; g School of Computer Science and Technology, Huazhong University of Science and Technology, Wuhan, Hubei, China

**Keywords:** Atractylenolide I, ATP6V0D2, clear cell renal cell carcinoma, HIF2α, autophagic degradation

## Abstract

Clear cell renal cell carcinoma (ccRCC) is tightly associated with *VHL* (von Hippel-Lindau tumor suppressor) mutation and dysregulated angiogenesis. Accumulating evidence indicates that antiangiogenic treatment abolishing tumor angiogenesis can achieve longer disease-free survival in patients with ccRCC. Atractylenolide I (ATL-I) is one of the main active compounds in *Atractylodes macrocephala* root extract and exhibits various pharmacological effects, including anti-inflammatory and antitumor effects. In this study, we revealed the potent antitumor activity of ATL-I in ccRCC. ATL-I exhibited robust antiangiogenic capacity by inhibiting EPAS1/HIF2α-mediated VEGFA production in VHL-deficient ccRCC, and it promoted autophagic degradation of EPAS1 by upregulating the ATPase subunit ATP6V0D2 (ATPase H+ transporting V0 subunit d2) to increase lysosomal function and facilitated fusion between autophagosomes and lysosomes. Mechanistically, ATP6V0D2 directly bound to RAB7 and VPS41 and promoted the RAB7-HOPS interaction, facilitating SNARE complex assembly and autophagosome-lysosome fusion. Moreover, ATP6V0D2 promoted autolysosome degradation by increasing the acidification and activity of lysosomes during the later stages of macroautophagy/autophagy. Additionally, we found that ATL-I could decrease the level of EPAS1, which was upregulated in sunitinib-resistant cells, thus reversing sunitinib resistance. Collectively, our findings demonstrate that ATL-I is a robust antiangiogenic and antitumor lead compound with potential clinical application for ccRCC therapy.

**Abbreviations**: ATL-I: atractylenolide I; ATP6V0D2: ATPase H+ transporting V0 subunit d2; CAM: chick chorioallantoic membrane; ccRCC: clear cell renal cell carcinoma; CTSB: cathepsin B; CTSD: cathepsin D; GO: Gene Ontology; HIF-1: HIF1A-ARNT heterodimer; HOPS: homotypic fusion and protein sorting; KDR/VEGFR: kinase insert domain receptor; KEGG: Kyoto Encyclopedia of Genes and Genomes; RCC: renal cell carcinoma; SNARE: soluble N-ethylmaleimide-sensitive factor attachment protein receptor; TCGA: The Cancer Genome Atlas; TEM: transmission electron microscopy; TKI: tyrosine kinase inhibitor; V-ATPase: vacuolar-type H±translocating ATPase; VEGF: vascular endothelial growth factor; VHL: von Hippel-Lindau tumor suppressor.

## Introduction

Renal cell carcinoma (RCC) is recognized as a highly aggressive malignancy within the urological oncology spectrum, with a concerning trend of rising incidence and mortality rates [1,2]. Surgical resection is the primary treatment modality for RCC diagnosed before metastatic spread. However, up to 30% of patients present regional or distant metastases at initial diagnosis [3]. Pathological neo-vascularization is a hallmark of clear cell renal cell carcinoma (ccRCC) [4]. ccRCC, the predominant form of RCC that constitutes approximately 85% of all RCC cases, has a strong genetic link to mutations in the *VHL* (von Hippel-Lindau tumor suppressor) gene and is characterized by the dysregulation of angiogenesis [4–6]. A common feature observed in many solid tumors, including ccRCC, is the overexpression (oe) of VEGF (vascular endothelial growth factor) and its receptor KDR/VEGFR (kinase insert domain receptor), which play a pivotal role in tumor angiogenesis [7]. These facts have led to several antiangiogenic agents targeting either VEGF or KDR. Tyrosine kinase inhibitors (TKIs), represented by sunitinib, exert an antiangiogenic effect through the inhibition of multiple targets, including KDR, and are well-established first-line treatments for metastatic RCC [8,9]. However, TKIs may upregulate VEGF pathway members to compensate for the loss of upstream KDR signaling, which, in turn, is involved in resistance to TKI treatment [10,11]. Studies have shown that nearly 20% of patients are primarily resistant to TKI therapy, and almost all patients may develop acquired resistance after receiving 6–15 months of TKI therapy [12]. Meanwhile, anti-VEGF antibodies, such as bevacizumab, are too high in molecular weight to penetrate solid tumor tissues [13]. Moreover, antiangiogenic therapy is limited by serious side effects, such as hypertension and renal toxicity [14,15]. Therefore, novel antiangiogenic medicines, especially small compounds with low toxicity, are urgently needed for patients with ccRCC.

Plant natural products are rich in compounds with diverse biological functions. Some extracts have antitumor pharmacological effects and can act as regulators of drug resistance [16,17]. Atractylenolide I (ATL-I) is one of the main active compounds in *Atractylodes macrocephala* root extract, with various pharmacological effects, including anti-inflammatory and antitumor effects [18,19]. A recent study showed that ATL-I can enhance the antitumor effect of cabozantinib on prostate cancer by targeting the Hsp27 signaling pathway [20]. In addition, by activating tumor antigen presentation, ATL-I can enhance the efficacy of immune checkpoint blockade therapies [21]. However, whether ALT-1 has antitumor and antiangiogenic effects on RCC remains largely unclear, and further investigations are needed to determine the potential anticancer effects of ATL-I.

In the present study, we revealed the potent antitumor and antiangiogenic activity of ATL-I in VHL-deficient ccRCC. Mechanistically, ATL-I promoted the autophagic degradation of EPAS1/HIF2α by upregulating ATP6V0D2 (ATPase H+ transporting V0 subunit d2) to increase lysosomal function and facilitated the fusion of autophagosomes and lysosomes. In addition, our investigation revealed that ATL-I is capable of reducing the expression of EPAS1 and reversing the resistance to sunitinib, thereby indicating its promising potential as a novel therapeutic agent for the treatment of ccRCC.

## Results

### ATL-I inhibits the proliferation, migration, and invasion of RCC cells

**Figure 1.**
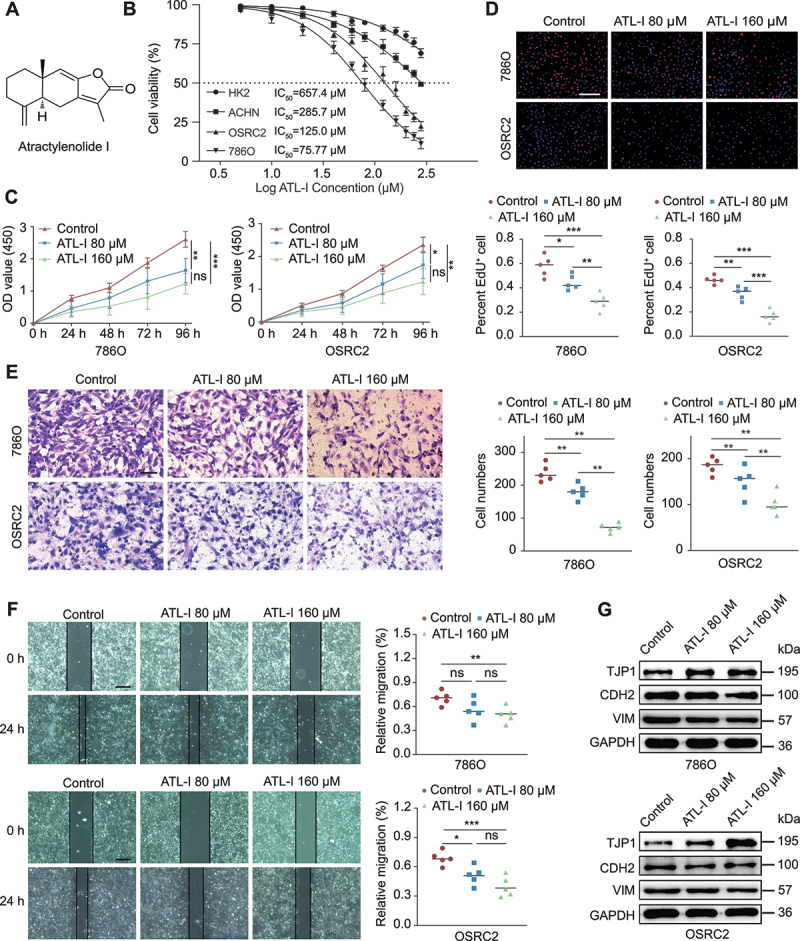
ATL-I inhibits RCC proliferation, invasion, and migration in a dose-dependent manner. (A) chemical structure of ATL-I. (B) after treating HK-2, ACHN, 786O, and OSRC2 cells with various concentrations of ATL-I for 36 h, cell viability in each group was measured using the CCK-8 assay. Dose-response curves were plotted, and half-maximal inhibitory concentrations values were calculated. (C) effect of ATL-I on 786O and OSRC2 cell viability at different time points (0 to 96 h). Scale bar: 100 μm. (D) after treatment with 0, 80, or 160 μM ATL-I for 48 h, cell proliferation was determined by EdU assay. (E) transwell assays were performed to evaluate cell invasion after incubation with 0, 80, or 160 μM ATL-I for 48 h. Scale bar: 50 μm. (F) cells were treated with different concentrations of ATL-I for 24 h, and wound healing assays were performed to evaluate cell migration. Scale bar: 100 μm. (G) changes in EMT markers between the control and ATL-I treatment groups. (*p < 0.05, **p < 0.01, ***p < 0.001.).

Atractylenolide, a compound extractable from the *Atractylodes macrocephala*, is known to have three distinct variants: ATL-I, ATL-II, and ATL-III. Among these, ATL-I has been recognized for its superior antitumor efficacy (Figure 1A). Our initial assessment focused on the impact of ATL-I on the proliferation of human renal tubular epithelial cells (HK-2) and three RCC cell lines (ACHN, 786O, and OSRC2). Following a 48-h exposure to escalating doses of ATL-I, the cell viability was determined using the Cell Counting Kit-8 (CCK-8) assay. The calculated half-maximal inhibitory concentrations for HK2, ACHN, OSRC2, and 786O were 657.4 μM, 285.7 μM, 125.0 μM, and 75.77 μM, respectively (Figure 1B). ATL-I exhibited a relatively weak inhibitory influence on ACHN cells, in contrast to its more pronounced suppressive effect on 786O and OSRC2 cells (Figure 1C, Figure S1A and B). It was noteworthy that the effect of ATL-I on HK2 cells was mild, with only a modest reduction on cell proliferation observed at higher concentrations (Figure S1B). The results from colony formation assays further corroborated the modest inhibitory impact of ATL-I on HK2 cell proliferation (Figure S1C). To further investigate the antitumor activity of ATL-I, cells were treated with ATL-I for 48 h and subjected to EdU assays. Our observations indicated that ATL-I significantly impeded the proliferation of 786O and OSRC2 cells (Figure 1D and S1D). In contrast, ACHN cell proliferation was only inhibited at higher ATL-I concentrations (160 μM) (Figure S1E). According to the above results, ATL-I showed more selective cytotoxic effects on RCC cells, particularly ccRCC cells, than on normal renal tubular epithelial cells. Subsequently, we proceeded to assess the influence of ATL-I on the invasive and migratory capabilities of RCC cells. Utilizing wound healing and Matrigel transwell assays, we observed that ATL-I significantly attenuated the migration and invasion abilities of 786O and OSRC2 cells (Figure 1E,F Figure S1F), while the inhibitory effect on ACHN cells was relatively weak (Figure S1H and I). Additionally, we detected changes in markers associated with mesenchymal-epithelial transition. The administration of ATL-I resulted in a considerable reduction in the levels of CDH2/N-cadherin and VIM (vimentin) while concurrently leading to an increase in the expression of TJP1/ZO-1 (Figure 1G and S1G).

### ATL-I induces cell cycle arrest and apoptosis

**Figure 2.**
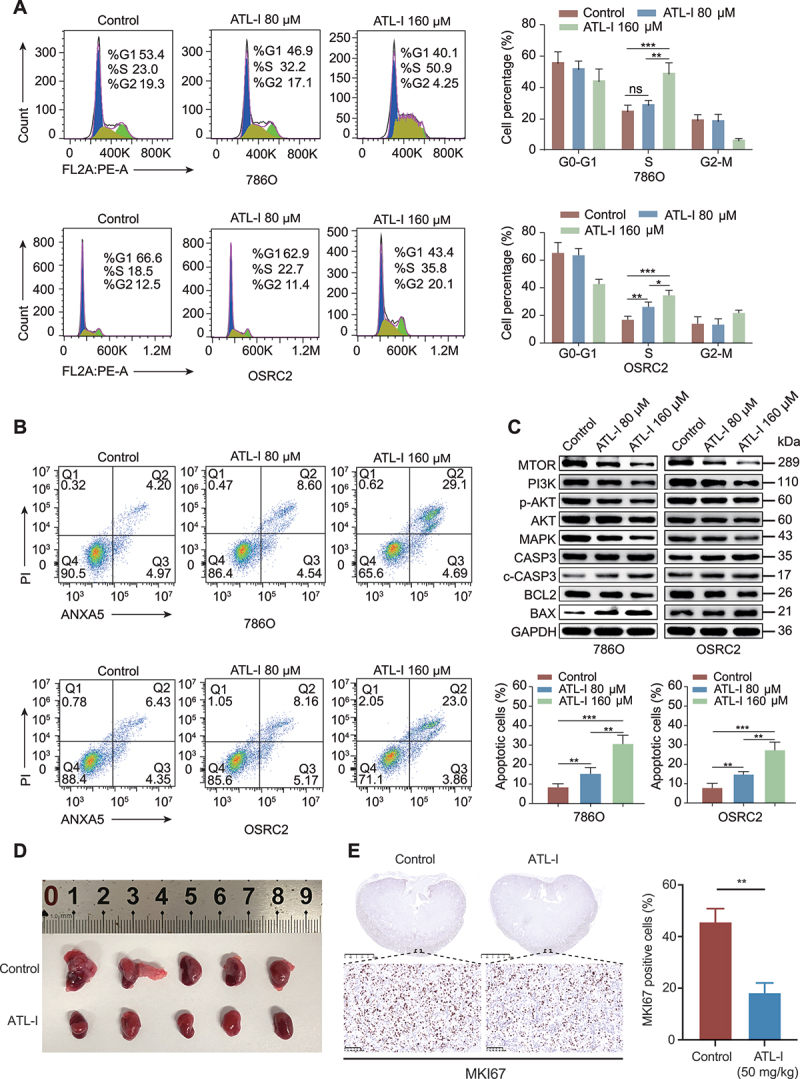
ATL-I suppresses cell cycle progression and induces apoptosis in ccRCC. (A and B) cell cycle distribution and apoptosis rates were determined by flow cytometry. (C) the expression of PI3K, AKT, p-akt, MTOR, and apoptosis-related markers in the control and ATL-I groups. (D) establishment of an orthotopic xenograft model using wild-type 786O cells, followed by a four-week treatment with ATL-I (50 mg/kg/day, i.P.). Anatomical images of orthotopic xenografts from the two groups. (E) immunohistochemical analysis of MKI67 expression in tumors from the two groups was performed, followed by statistical analysis. Scale bar: 2.5 mm or 100 μm (*p < 0.05, **p < 0.01, ***p < 0.001.).

Previous research has demonstrated that ATL-I potently inhibits the proliferation of bladder cancer cells, primarily through inducing cell cycle arrest at the G_2_/M transition [22]. Building on this foundation, we investigated the potential link between the growth-inhibitory effects of ATL-I and cell cycle modulation. Our findings revealed that treatment with ATL-I led to a dose-dependent increase of 786O and OSRC2 cells in the S phase, accompanied by a corresponding decrease in the proportion of cells in the G_0_-G_1_ and G_2_-M phases (Figure 2A). Additionally, we discerned that ATL-I could trigger apoptosis in a dose-dependent fashion (Figure 2B). This was evidenced by a significant upregulation of the pro-apoptotic proteins, cleaved CASP3 (caspase 3) and BAX, in cells treated with ATL-I, as opposed to the control group. Conversely, the expression of the anti-apoptotic protein BCL2 was markedly diminished in the ATL-I-treated cells (Figure 2C). Given the established role of ATL-I as a traditional Chinese medicine with documented influence on the phosphoinositide 3-kinase (PI3K)-AKT-MTOR, MAPK, and NFKB/NF-κB signaling pathways [21,23–25], our study aimed to estimate the modulation of key proteins within these pathways. Our analysis confirmed the findings of earlier investigations, showing a significant downregulation of MTOR, PI3K, phosphorylated AKT, and MAPK protein levels in the ATL-I-treated group (Figure 2C).

### ATL-I inhibits the proliferation of ccRCC in vivo

Following the promising in vitro findings, we advanced to in vivo studies by establishing a subcutaneous xenograft tumor model in female athymic nude mice using 786O cells. The mice received four-week treatments of ATL-I at dosages of either 25 mg/kg or 50 mg/kg. It was observed that the lower dosage of 25 mg/kg exhibited a moderate inhibitory effect on tumor growth, whereas the higher dosage of 50 mg/kg yielded a significantly enhanced suppression (Figure S2A). Additionally, the expression of MKI67/Ki-67 was evaluated in the tumor tissues. A substantial decrease in MKI67 levels was noted within the ATL-I treated groups (Figure S2B). To assess the therapeutic efficacy of ATL-I more accurately in a setting that closely resembles the biological and clinical context of ccRCC, we established an orthotopic xenograft model. Upon necropsy, the group treated with ATL-I (50 mg/kg) exhibited a notable decrease in tumor size compared to that of the control group (Figure 2D and S2C). Correspondingly, there was a significant decline in MKI67 expression following ATL-I treatment (Figure 2E). To further assess the toxicity of ATL-I, we consistently monitored the body weights of the mice throughout the treatment period. The data indicated no significant variation in the average body weight between the treated and control groups (Figure S2D). Furthermore, a histopathological examination of the heart, liver, spleen, lung, and kidney tissues from both groups was conducted. The results demonstrated no significant differences in the histomorphology of these organs, suggesting that ATL-I has a favorable safety profile in vivo (Figure S2E).

### Anti-angiogenic effects of ATL-I in vivo

**Figure 3.**
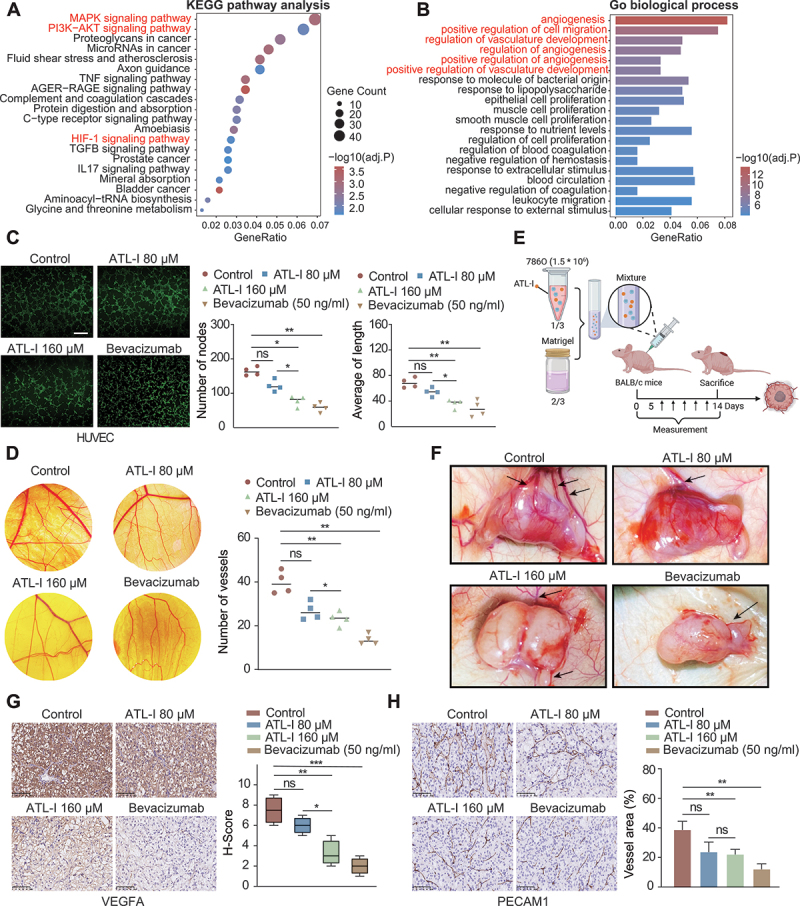
ATL-I inhibits angiogenesis in vitro and in vivo. (A and B) KEGG pathway and GO enrichment analyses of genes differentially expressed upon ATL-I treatment. (C) the effects of ATL-I on vegf-induced tube formation in HUVECs, the number of branch nodes and the length of the tube networks were quantified and compared. Scale bar: 100 μm (D) ATL-I inhibited neo-vascular formation in the CAM assay. (E and F) Representative images of Matrigel plug assays following DMSO or ATL-I treatment. (G) VEGFA IHC staining in control and ATL-I-treated Matrigel plugs. Quantification of vegfa-positive cells. Scale bar: 100 μm. In the present set of experiments, bevacizumab was used as the positive control. (H) PECAM1 IHC staining in control and ATL-I-treated Matrigel plugs. The percent vessel area was quantified and compared. Scale bar: 100 μm. (*p < 0.05, **p < 0.01, ***p < 0.001.).

To further explore the antitumor mechanisms of ATL-I, we conducted a comprehensive transcriptome analysis comparing the gene expression profiles of the ATL-I treatment and control groups. Kyoto Encyclopedia of Genes and Genomes (KEGG) and Gene Ontology (GO) analysis were performed based on the differential expressed genes between these two groups. Utilizing the KEGG pathway analysis, we identified a significant correlation between ATL-I treatment and multiple cancer-related signaling pathways, such as the MAPK, PI3K-AKT, and HIF1A/HIF1α (hypoxia inducible factor 1 subunit alpha)-ARNT/HIF1β (aryl hydrocarbon receptor nuclear translocator) heterodimer (HIF-1) signaling pathways. HIF-1 serves as a pivotal transcription factor, acting as the central regulator of oxygen homeostasis. Under hypoxic conditions, HIF-1 orchestrates the expression of a broad spectrum of hypoxia-inducible genes, thereby ensuring cellular adaptation and survival. Interestingly, the HIF1A-EPAS1 pathway has not been previously reported to be associated with ATL-I (Figure 3A). GO analysis further indicated that ATL-I treatment is intimately linked to angiogenesis, particularly in the regulation of vasculature development (Figure 3B). To validate these in silico findings, we investigated the anti-angiogenic effects of ATL-I both in vitro and in vivo. We observed that ATL-I exhibited stronger activity in preventing HUVEC tube formation at 160 μM, with a similar effect to that produced by bevacizumab (50 ng/ml) (Figure 3C). Furthermore, the results of two angiogenesis models, including the chick chorioallantoic membrane (CAM) and Matrigel plug assays, substantiated the anti-angiogenic ability of ATL-I in vivo. The CAM assays confirmed that ATL-I could effectively suppress the formation of neo-angiogenesis (Figure 3 3D and S3A). In the Matrigel plug assay, Matrigel mixed with 786O cells, with or without ATL-I, was subcutaneously implanted into mice (Figure 3E). The plugs containing ATL-I showed a significant reduction in angiogenesis (Figure 3F and S3B). IHC staining revealed that ATL-I treatment led to a pronounced decrease in VEGFA production (Figure 3G), which is a critical driver of tumor angiogenesis. This reduction in VEGFA expression was associated with a substantial inhibition of tumor angiogenesis, as indicated by the diminished PECAM1/CD31 staining (Figure 3H and S3C). In addition, we evaluated PECAM1 levels in both subcutaneous and orthotopic tumor tissues. Notably, within the ATL-I treated group, a significant decrease in PECAM1 expression was observed (Figure S3D and E).

### ATL-I-induced ATP6V0D2 upregulation accelerates the degradation of EPAS1 by the autophagy pathway

**Figure 4.**
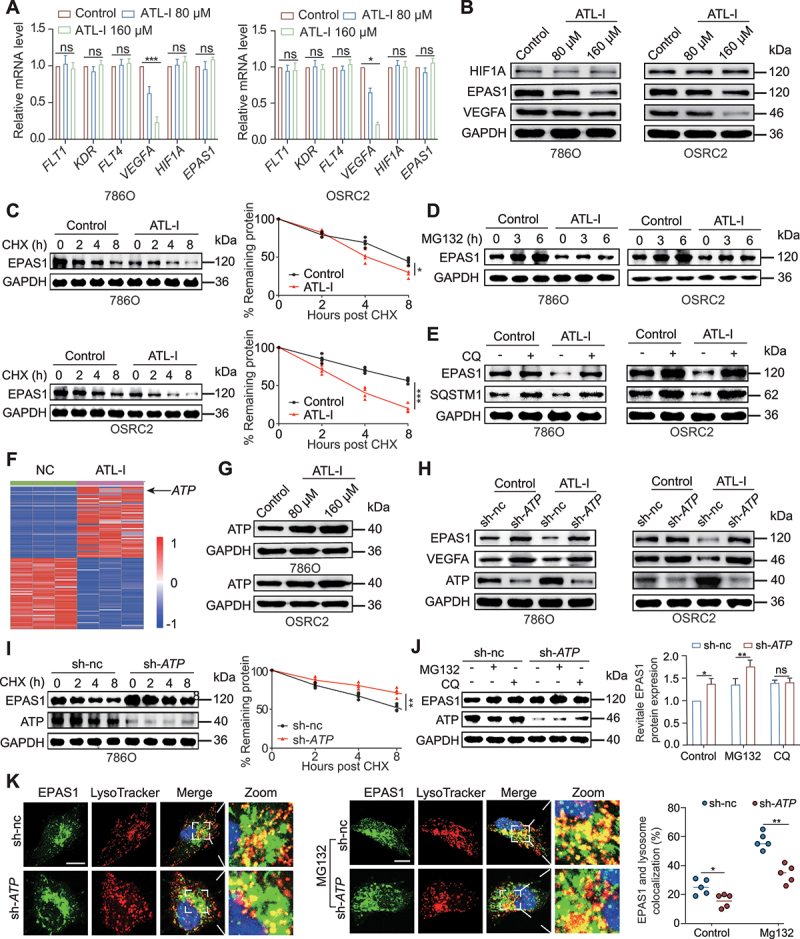
Upregulation of ATP6V0D2 by ATL-I accelerated the autophagic degradation of EPAS1. (A) expression detection of *HIF1A*, *EPAS1*, *VEGFA*, *FLT1/VEGFR1*, *KDR/VEGFR2*, and *FLT4/VEGFR3* by rt-qPCR in 786O and OSRC2 cells treated with 0, 80, and 160 μM ATL-I for 48 h. (B) Western blot was performed to detect the protein levels of HIF1A, EPAS1 and VEGFA in 786O and OSRC2 cells that were treated with 0, 80, or 160 μM ATL-I for 72 h. (C) 786O and OSRC2 cells were untreated or treated with 80 μM ATL-I for 48 h. After washing with PBS, the cells were incubated with 20 µg/ml cycloheximide and collected at the indicated times. EPAS1 protein levels were determined by western blot. (D) ccRCC cells were untreated or treated with ATL-I for 48 h. Immunoblotting was performed to detect EPAS1 protein levels in the two groups of cells incubated with MG132 (20 μM) for the indicated times. (E) ccRCC cells were untreated or treated with ATL-I for 48 h. Immunoblotting was performed to detect EPAS1 protein levels in the two groups of cells that were not incubated or incubated with chloroquine (10 μM) for 12 h. (F) Heatmap showing the top 50 upregulated and downregulated genes between the control and ATL-I treatment groups. (G) protein levels of ATP6V0D2 in 786O and OSRC2 cells treated with different concentrations of ATL-I. (H) immunoblotting was performed to detect the expression of EPAS1 and VEGFA in sh-nc and sh-*ATP6V0D2* ccRCC cells with or without ATL-I treatment. (I) 786O cells with an expression vector or *ATP6V0D2* knockdown were treated with 20 µg/ml cycloheximide at the indicated times. EPAS1 and ATP6V0D2 protein levels were determined by immunoblotting. (J) 786O cells with an expression vector or *ATP6V0D2* knockdown were treated with 10 μM chloroquine or 20 μM MG132 for 8 h. EPAS1 and ATP6V0D2 protein levels were detected by western blot. (K) 786O cells, expression vector or knockdown of *ATP6V0D2*, were unincubated or incubated with 20 μM MG132 for 6 h. The cells were stained with anti-EPAS1/HIF2α and LysoTracker. The colocalization of EPAS1 and lysosomes in the cytoplasm was quantified. Scale bar: 10 μm. (*p < 0.05, **p < 0.01, ***p < 0.001.).

It is well known that ccRCC is frequently characterized by inactivating mutations in *VHL*, which lead to EPAS1-mediated VEGFA production and the development of highly vascularized tumors [24–27]. Thus, we compared the mRNA levels of HIF1A-EPAS1-VEGFA-KDR signaling pathway components between the control and ATL-I groups. As shown in Figure 4A, ATL-I strongly repressed *VEGFA* mRNA transcription in 786O and OSRC2 cells. However, *HIF1A/HIF1α* and *EPAS1* mRNA expression levels were similar between the ATL-I treatment and control groups (Figure 4A). Considering that HIF1A and EPAS1 play a key role in VEGFA production [24,28], we further detected HIF1A and EPAS1 protein expression in the control and ATL-I groups. The results demonstrated that EPAS1 protein expression was significantly decreased in the ATL-I group, suggesting that ATL-I might regulate EPAS1 expression via post-translational modifications (Figure 4B). However, for the papillary RCC cell line ACHN with no *VHL* mutation and low expression of EPAS1, ATL-I treatment produced a relatively modest change in the mRNA and protein expression levels of VEGFA (Figure S4A and B). Moreover, ATL-I treatment shortened the half-life of EPAS1 proteins in 786O cells, which was consistent with the findings in OSRC2 cells (Figure 4C). These results suggested that ATL-I treatment might cause VEGFA downregulation by accelerating EPAS1 degradation in ccRCC. In light of the fact that both the ubiquitin-proteasome system and lysosomal proteolysis-mediated pathway are capable of facilitating the degradation of the EPAS1 protein, our subsequent analysis focused on assessing the effects of proteasome and lysosomal inhibitors on the regulation of EPAS1 protein expression by ATL-I [29]. Western blot showed that EPAS1 protein expression significantly decreased in the ATL-I group compared with the control group in the presence of MG132 (Figure 4D), suggesting that another proteasome-independent degradation pathway may participate in ATL-I-mediated EPAS1 degradation. Thus, we incubated 786O and OSRC2 cells in the presence of the lysosomal inhibitor chloroquine and observed similar levels of EPAS1 and SQSTM1/p62 expression between the control and ATL-I groups (Figure 4E). To further verify the role of autophagy in this process, we investigated the protein expression of EPAS1 in 786O cells after knocking down *ATG5* to inhibit autophagic activity. The results indicated that knocking down *ATG5* can reverse the inhibitory effect of ATL-I on EPAS1 (Figure S4C), which indicates that ATL-I mediates EPAS1 degradation by promoting the autophagy pathway. Given the intricate regulatory processes governing autophagy, inhibition of the MTOR signaling pathway can activate autophagy [30,31]. Our preceding results demonstrated that ATL-I substantially impedes the MTOR signaling pathway. To further investigate whether ATL-I influences cellular autophagy via alternate routes, both the control and ATL-I-treated groups were exposed to the MTOR inhibitor rapamycin. Western blot analysis revealed that, in comparison to those in the control group, there were marked reductions in SQSTM1 and EPAS1 expression in cells treated with rapamycin alone. Intriguingly, cells subjected to combined treatment with rapamycin and ATL-I presented a more significant decrease in the protein levels of SQSTM1 and EPAS1 (Figure S4D). These findings imply that ATL-I may facilitate autophagy through mechanisms other than MTOR pathway inhibition.

To further investigate the mechanism by which ATL-I regulates EPAS1 autophagic degradation, we screened autophagy-related differentially expressed genes in the RNA-seq dataset. *ATP6V0D2*, which was the second most upregulated gene after ATL-I treatment, attracted our attention because it has been reported to be associated with lysosomal function [32,33] (Figure 4F and S4E). Dose-dependent ATL-I-induced upregulation of ATP6V0D2 was confirmed by RT-qPCR and western blot analysis in 786O and OSRC2 cells (Figure 4G and S4F). Interestingly, ATP6V0d2 has been reported to target EPAS1 for lysosome-mediated degradation in macrophages [34]. Therefore, it is plausible to hypothesize that ATL-I accelerates the degradation of EPAS1 by activating ATP6V0D2. We observed that *ATP6V0D2* deletion largely reversed the downregulation of EPAS1 and VEGFA expression induced by ATL-I (Figure 4H). The cycloheximide chase assay further substantiated that downregulation of ATP6V0D2 enhanced EPAS1 protein stability (Figure 4I). Chloroquine treatment eliminated the ATP6V0D2 depletion-induced increase in EPAS1 protein expression (Figure 4J). Next, we used LysoTracker Red to label lysosomes and investigated whether ATP6V0D2 can affect the localization of EPAS1 to lysosomes. After using MG132 to block the proteasome degradation pathway, the colocalization of EPAS1 and lysosomes was enhanced. However, compared with the control group, *ATP6V0D2* knockdown largely reduced the colocalization of EPAS1 and lysosomes (Figure 4K). Finally, CCK-8 and cell apoptosis assays were performed to verify whether *ATP6V0D2* knockdown could rescue the inhibitory effect of ATL-I treatment on ccRCC proliferation. The results showed that the inhibition of cell viability and the proapoptotic effect of ATL-I were significantly recovered by *ATP6V0D2* knockdown (Figure S4G and H).

### ATP6V0D2 inhibited ccRCC proliferation, invasion, and metastasis

**Figure 5.**
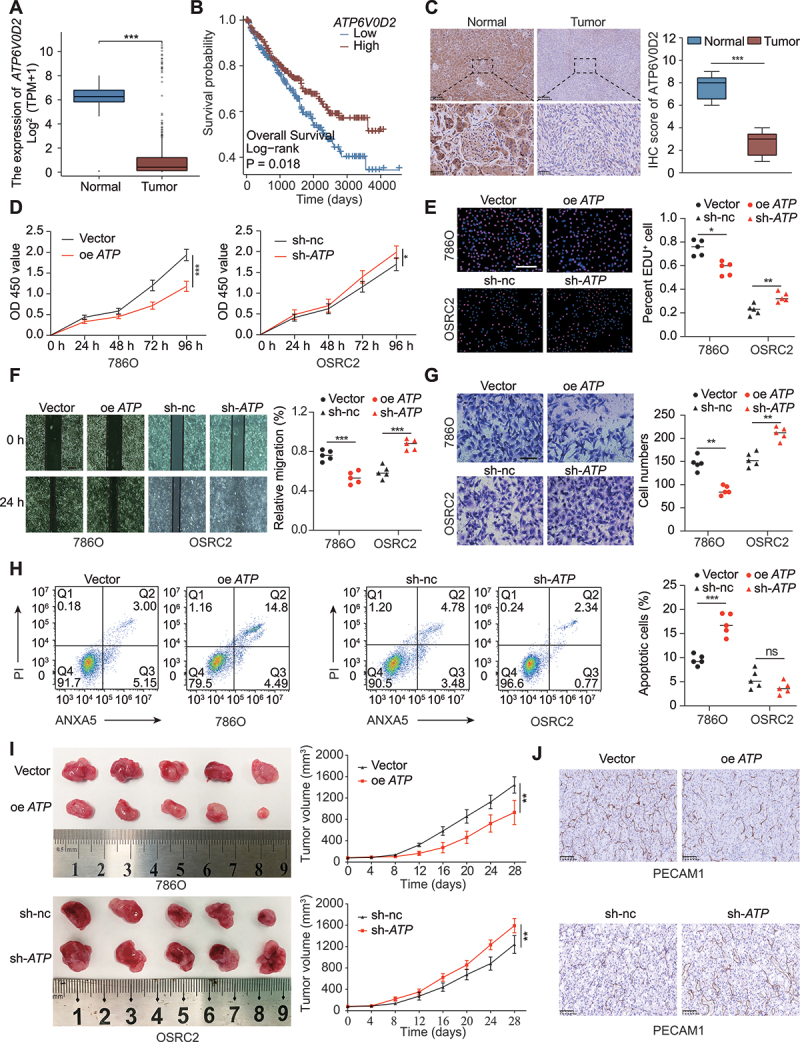
ATP6V0D2 inhibited RCC proliferation, invasion, and metastasis. (A) Expression of *ATP6V0D2* between ccRCC tumor and normal tissues in the TCGA cohort. (B) Differences in overall survival between *ATP6V0D2*^high^ and *ATP6V0D2*^low^ patients. (C) IHC analysis of ATP6V0D2 expression in tumor-adjacent tissues and tumor tissues. Scale bar: 50 or 250 μm. (D and E) cell proliferation was assessed using CCK-8 and EdU assays in the vector and oe *ATP6V0D2* groups, as well as in the sh-nc and sh-*ATP6V0D2* groups. Scale bar: 100 μm. (F and G) cell invasion and migration were evaluated using Transwell invasion and wound healing tests in the vector and oe *ATP6V0D2* groups, as well as in the sh-nc and sh-*ATP6V0D2* groups. Scale bar: 100 or 50 μm. (H) The percentage of apoptotic cells in the vector, oe *ATP6V0D2*, sh-nc and sh-*ATP6V0D2* groups. (I) Growth rates of xenografts between the vector and oe *ATP6V0D2* groups and the sh-nc and sh-*ATP6V0D2* groups. (J) IHC staining for PECAM1 in xenografts from the vector, oe *ATP6V0D2*, sh-nc and sh-*ATP6V0D2* groups. Scale bar: 100 μm. (*p < 0.05, **p < 0.01, ***p < 0.001.).

Data retrieved from The Cancer Genome Atlas (TCGA) database revealed a significant downregulation of *ATP6V0D2* expression in patients with ccRCC (Figure 5A). Patients with diminished *ATP6V0D2* expression exhibited notably poorer overall survival, progression-free survival, and disease-specific survival compared to those with high expression levels (Figure 5B and S5A). Moreover, *ATP6V0D2* expression was found to decrease significantly with the advancement of both TNM and pathological stages (Figure S5B-E). IHC analysis of protein expression in twelve pairs of tumors and peritumor tissues confirmed a substantial reduction of ATP6V0D2 in the tumor tissues (Figure 5C). Functional assays demonstrated that overexpression of *ATP6V0D2* in 786O cells led to a significant reduction in cell proliferation, migration, and invasion, whereas knockdown of *ATP6V0D2* yielded the converse effects (Figure 5D-G). Additionally, *ATP6V0D2* overexpression increased the 786O cell apoptosis rate by 10% (Figure 5H). Moreover, these findings were validated in vivo. The overexpression of *ATP6V0D2* resulted in a considerable reduction in tumor volume, while the knockdown of *ATP6V0D2* led to an increase in tumor volume (Figure 5I). IHC for PECAM1 staining suggested that the number of blood vessels decreased in the oe *ATP6V0D2* group and increased in the sh-nc group (Figure 5J). We also detected the impact of ATP6V0D2 on ACHN, a papillary RCC cell line with no *VHL* mutation and low expression of EPAS1. We found that *ATP6V0D2* overexpression had a weaker effect on ACHN cell viability under normoxic conditions. However, an increase in ATP6V0D2 strongly inhibited ACHN cell viability under hypoxic conditions (Figure S5F). Western blot revealed that upregulation of ATP6V0D2 decreased hypoxia-induced EPAS1 expression (Figure S5G). Taken together, the above results suggested that ATP6V0D2 suppressed the progression of RCC by modulating EPAS1 expression.

### ATL-I promotes autophagosome-lysosome fusion and lysosomal function mediated by ATP6V0D2

**Figure 6.**
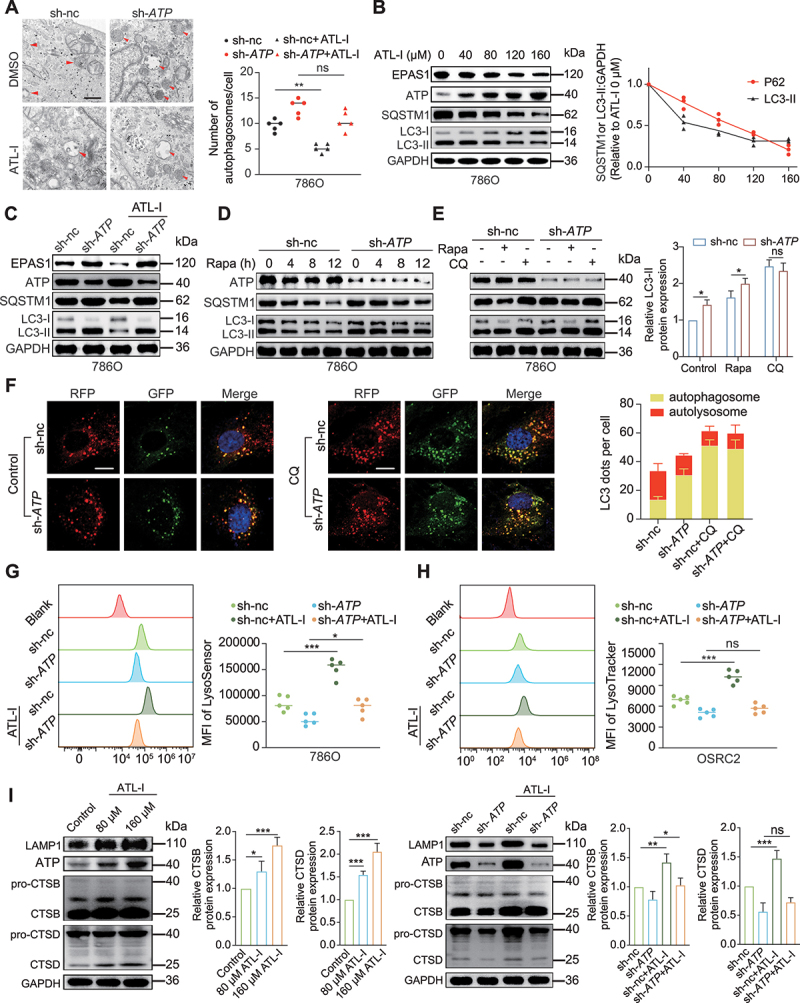
ATL-I promotes autophagosome-lysosome membrane fusion and lysosomal function through ATP6V0D2. (A) Autophagosome detection by transmission electron microscopy in sh-nc and sh-*ATP6V0D2* cells untreated or treated with 80 µm ATL-I. Scale bar: 0.5 μm. (B) Immunoblot analysis of SQSTM1, LC3-II, ATP6V0D2, and EPAS1 in 786O cells treated with various concentrations of ATL-I. (C) Immunoblot analysis of SQSTM1, LC3-II, ATP6V0D2, and EPAS1 in sh-nc and sh-*ATP6V0D2* cells untreated or treated with 80 µm ATL-I. (D) Immunoblot analysis of SQSTM1, LC3-II, and ATP6V0D2 in sh-nc and sh-*ATP6V0D2* cells that were either untreated or treated with 50 nM rapamycin at the indicated times. (E) 786O cells with an expression vector or *ATP6V0D2* knockdown were treated with 50 nM rapamycin and 10 μM chloroquine for 8 h. SQSTM1, LC3-II, and ATP6V0D2 protein levels were detected by immunoblotting. (F) sh-nc and sh-*ATP6V0D2* 786O cells that were transduced with RFP-GFP-LC3-expressing lentivirus. Cells were untreated or treated with 10 μM chloroquine for 4 h. Fluorescence analysis of the mean number of RFP and GFP puncta. Scale bar: 10 μm. (G) Lysosomal acidification was evaluated by flow cytometry with LysoSensor staining in sh-nc and sh-*ATP6V0D2* cells untreated or treated with 80 µm ATL-I. (H) Lysosomal activity was evaluated by flow cytometry with LysoTracker staining in sh-nc and sh-*ATP6V0D2* cells untreated or treated with 80 µm ATL-I. (I) immunoblot analysis of LAMP1, CTSB, and CTSD in 786O cells treated with different concentrations of ATL-I and in sh-nc and sh-*ATP6V0D2* cells untreated or treated with 80 µm ATL-I. (*p < 0.05, **p < 0.01, ***p < 0.001.).

Transmission electron microscopy (TEM) analysis revealed a significant reduction in the number of autophagosomes per cell after ATL-I treatment compared to that in the control group. In contrast, the opposite results were found when *ATP6V0D2* was knocked down (Figure 6A and S6A). Western blot analysis indicated that ATL-I had a suppressive effect on the expression levels of LC3-II and SQSTM1 (Figure 6B). Nevertheless, the deletion of *ATP6V0D2* significantly counteracted this impact (Figure 6C and S6B). To further substantiate this point, sh-nc and sh-*ATP6V0D2* cells were subjected to rapamycin treatment to induce autophagy. Consistently, SQSTM1 and LC3-II were quickly degraded over time in the sh-nc group but did not change much in the sh-*ATP6V0D2* group (Figure 6D). Rapamycin enhanced LC3-II in both sh-nc and sh-*ATP6V0D2* cells. However, after chloroquine treatment, the levels of SQSTM1 and LC3-II were similar between the two groups (Figure 6E). The above results indicated that ATP6V0D2 promoted autophagic flux without affecting the formation of autophagosomes. Furthermore, we performed a detailed examination of LC3 localization by using RFP-GFP-tagged LC3. The fluorescence of GFP is suppressed by the acidic pH of lysosomes, enabling the distinction between autophagosomes (GFP^+^ and RFP^+^: yellow) and autolysosomes (GFP^−^ and RFP^+^: red). Knockdown of *ATP6V0D2* resulted in an increase in the number of RFP^+^-GFP^+^-LC3 yellow puncta, suggesting that while ATP6V0D2 did not impede the formation of autophagosomes, yet it significantly influenced their degradation process (Figure 6F). Nevertheless, when autophagosome and lysosome fusion was blocked by chloroquine, the number of RFP^+^-GFP^+^-LC3 yellow puncta showed no statistically significant difference between the two groups (Figure 6F).

Considering that ATP6V0D2 is a component of the vacuolar H±ATPase (V-ATPase), we speculated that it may participate in V-ATPase-mediated lysosome acidification. We evaluated lysosomal acidification and activity using the lysosome-permeable fluorescent pH LysoSensor indicator and the lysosomotropic dye LysoTracker. The results showed that ATL-I increased lysosomal acidification and activity in a dose-dependent manner (Figure S6C and D). Silencing ATP6V0D2 abolished the increase in lysosomal acidity and activity induced by ATL-I (Figure 6G,H). CTSB (cathepsin B) and CTSD (cathepsin D) are two major lysosomal hydrolases, and their maturation requires lysosomal acidification [35,36]. We found that the increase in CTSB and CTSD following ATL-I treatment was largely reversed by *ATP6V0D2* knockdown (Figure 6I).

### ATP6V0D2 promotes the RAB7-HOPS interaction and facilitates SNARE complex assembly

**Figure 7.**
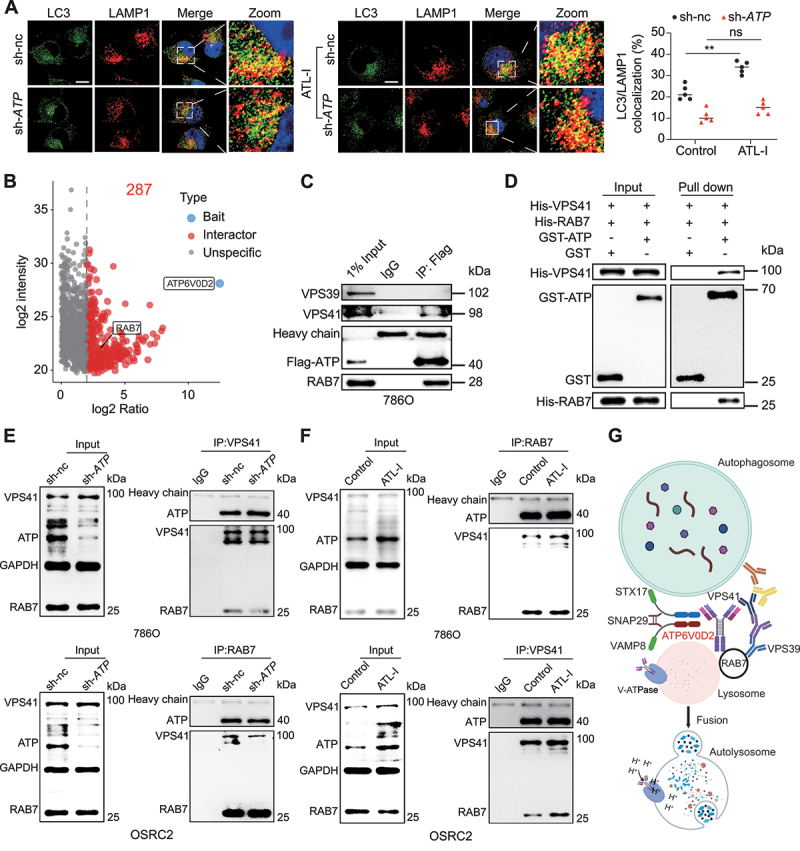
ATP6V0D2 promotes the RAB7-HOPS interaction and facilitates SNARE complex assembly. (A) colocalization of LC3 with LAMP1 examined by confocal microscopy in sh-nc and sh-*ATP6V0D2* cells untreated or treated with 80 µm ATL-I. Scale bar: 10 μm. (B) volcano plot of the mass spectrometry data. Coimmunoprecipitation and mass spectrometry were performed to identify ATP6V0D2-interacting proteins in 786O cells. RAB7 was the candidate among the ATP6V0D2-interacting proteins. (C) 786O cells stably expressing flag-ATP6V0D2 were immunoprecipitated by beads. Samples were immunoblotted for endogenous VPS41, VPS39, and RAB7. (D) GST, GST-ATP6V0D2, his-VPS41, and his-RAB7 were expressed in Rosetta bacteria. The purified proteins were subjected to GST affinity-isolation assays and subsequent western blot analysis. (E) sh-nc and sh-*ATP6V0D2* cells were immunoprecipitated with either anti-VPS41 or anti-RAB7. The samples were subjected to western blot and probed with antibodies as indicated. (F) cells were untreated or treated with 80 μM ATL-I for 48 h and immunoprecipitated with either anti-RAB7 or anti-VPS41. The samples were subjected to western blot and probed with antibodies as indicated. (G) scheme illustrating the role of ATP6V0D2 in contributing to autophagosome-lysosome membrane fusion and lysosomal function. (**p < 0.01.).

The completion of autophagy is regulated not only by lysosomal activity but also by the progression of autophagosome-lysosome fusion. Therefore, we also evaluated whether ATP6V0D2 participated in the fusion of autophagosomes and lysosomes. Confocal colocalization analysis of punctate autophagosomal labeling (LC3) and lysosomal labeling (LAMP1) revealed that the promoting role of ATL-I on autophagosome and lysosome fusion could also be reversed by *ATP6V0D2* knockdown (Figure 7A). To clarify how ATP6V0D2 blocks the fusion of autophagosomes and lysosomes, we analyzed ATP6V0D2-binding proteins via immunoprecipitation followed by mass spectrometry. We found a strong interaction between ATP6V0D2 and RAB7, which regulates cell membrane trafficking (Figure 7B and S7A). Studies have shown that RAB7, together with the homotypic fusion and protein sorting (HOPS) complex (VPS41 and VPS39), plays a crucial role in the process of anchoring autophagosomes to lysosomes, ultimately leading to the fusion of their membrane bilayers [37–39]. We further explored whether ATP6V0D2 interacted with RAB7 and HOPS (VPS41 and VPS39) during autophagosome-lysosome fusion. In 786O cells, Flag-tagged ATP6V0D2 was found to combine with RAB7 and VPS41 (Figure 7C). We cotransfected 293T cells with FLAG-tagged ATP6V0D2, His-tagged RAB7, and His-tagged VPS41. The results verified that ATP6V0D2 interacted with RAB7 and VPS41 again (Figure S7B). Furthermore, the direct interaction was corroborated through GST affinity-isolation assays (Figure 7D). Thus, whether ATP6V0D2 promotes RAB7-HOPS-mediated autophagosome-lysosome fusion was further investigated. Immunofluorescence analysis revealed that *ATP6V0D2* knockdown significantly reduced the colocalization of RAB7 and VPS41, while ATL-I markedly increased this colocalization (Figure S7C). Moreover, coimmunoprecipitation assays suggested that the knockdown of *ATP6V0D2* decreased the interaction between RAB7 and VPS41 (Figure 7E). Accordingly, ATL-I treatment markedly promoted this process (Figure 7F).

The fusion of autophagosomes and lysosomes is facilitated by a group of proteins known as N-ethylmaleimide sensitive factor attachment protein receptors (SNAREs), including STX17, SNAP29, and VAMP8, in addition to the HOPS complex. A previous study pointed out that ATP6V0D2 can bind to STX17 and VAMP8, promoting the completion of autophagy in macrophages [33]. In our study, we also discovered that ATL-I increased the interaction between STX17 and VAMP8 (Figure S7D). In contrast, *ATP6V0D2* knockdown significantly reduced this interaction (Figure S7E). Consistently, *ATP6V0D2* knockdown significantly reduced the colocalization of STX17 and VAMP8, while ATL-I increased this colocalization (Figure S7F). Based on these results, we propose a potential mechanism by which ATL-I promotes autophagy mediated by ATP6V0D2. Briefly, ATP6V0D2 binds to RAB7 and VPS41, acting as an important bridge to promote the RAB7-HOPS interaction to increase autophagosome-lysosome fusion. In addition, it also increased the interaction between STX17 and VAMP8, contributing to autophagosome-lysosome fusion. In addition, it promotes autolysosome degradation by increasing the acidification and activity of lysosomes (Figure 7G).

### ATL-I enhances sunitinib sensitivity in ccRCC via inhibition of the EPAS1 pathway

**Figure 8.**
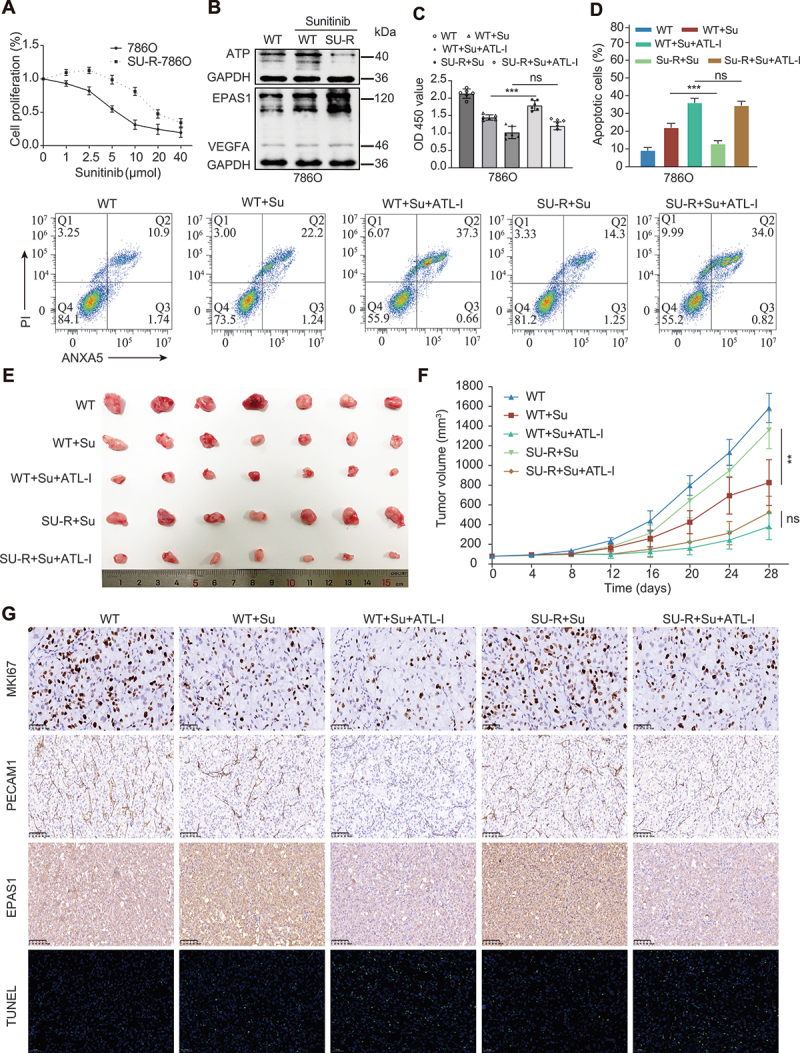
ATL-I enhances sunitinib sensitivity in ccRCC via inhibition of the EPAS1 pathway. (A) wildtype or sunitinib-resistant cells were treated with different concentrations of sunitinib for 48 h, and cell viability was measured by CCK-8 assay. (B) immunoblot analysis of EPAS1, VEGFA, and ATP6V0D2 in the indicated cells treated with or without sunitinib. (C and D) the indicated cells were treated with 5 μM sunitinib alone or in combination with 80 μM ATL-I for 36 h. Cell viability and apoptosis were determined by CCK-8 and flow cytometry assays. (E and F) 786O or SU-R-786O cells (1 × 10^6^) were injected subcutaneously into nude mice. The indicated five groups of mice were either treated with sunitinib (20 mg/kg) alone or treated with ATL-I (50 mg/kg) and sunitinib (20 mg/kg). Then, the mice were sacrificed, and the tumor volume was measured. (G) IHC staining showing the expression levels of MKI67, PECAM1, and EPAS1. Scale bar: 50 or 100 μm; TUNEL assay for cell apoptosis analysis in the five groups. Scale bar: 50 μm. (**p < 0.01, ***p < 0.001.).

Sunitinib, a multi-target tyrosine kinase inhibitor, has emerged as the primary treatment modality for patients with advanced RCC. Nevertheless, many findings have shown that sunitinib may trigger the overexpression of members of the VEGFA pathway, perhaps as a compensatory mechanism for the diminished upstream KDR signaling. This phenomenon has been implicated in the development of resistance to sunitinib therapy [26,40]. A sunitinib-resistant cell line, SU-R-786O, was generated in our study. Notably, SU-R-786O cells demonstrated a substantial increase in cell survival relative to wildtype 786O cells upon exposure to sunitinib (Figure 8A). Consistent with the findings of previous studies, EPAS1 and VEGFA were significantly upregulated in SU-R-786O cells compared with wildtype cells (Figure 8B). Intriguingly, the expression of ATP6V0D2 was remarkably downregulated in SU-R-786O cells (Figure 8B). ATL-I upregulated ATP6V0D2 expression, resulting in the degradation of EPAS1. We therefore explored whether ATL-I could reverse sunitinib resistance by reducing EPAS1 expression in ccRCC cells. CCK8 assays showed that SU-R-786O exhibited significantly increased cell viability compared with WT-786O after treatment with sunitinib. However, when combined with ATL-I treatment, there was no significant difference in sunitinib sensitivity between WT-786O and SU-R-786O cells (Figure 8C). The apoptosis assay results also confirmed the above findings (Figure 8D). Moreover, western blot indicated an increase in pro-apoptotic proteins (BAX and CASP3) and a decrease in EPAS1 and VEGFA (Figure S8A). Consistent with the in vitro results, combination treatment with ATL-I noticeably reversed the resistance of SU-R-786O cells to sunitinib (Figure 8E,F, Figure S8B). The combination groups exhibited a significant decrease in the MKI67-positive cells, the tumor vessel area, and the EPAS1 expression. In addition, there was a notable increase in the proportion of TUNEL-positive cells (Figure 8G and S8C). Taken together, these findings indicate that ATL-I can sensitize SU-R-786O cells to sunitinib via ATP6V0D2-mediated inhibition of the EPAS1-VEGFA pathway. ATL-I in combination with sunitinib exerts a synergistic effect in alleviating acquired sunitinib resistance.

## Discussion

Persistent pathological angiogenesis drives tumor progression and metastasis, especially in ccRCC [4,41]. The majority of ccRCCs are marked by the inactivation of the *VHL* gene, which leads to an aberrant regulation of hypoxia pathways. The *VHL* gene product plays a crucial role in the process of oxygen sensing by functioning as an E3 ubiquitin ligase. Its primary function is to target the HIF1A subunit of HIF-1 for degradation via the ubiquitin-proteasome system when oxygen levels are normal [11,25]. When the *VHL* gene is deleted or suppressed, there is an accumulation of HIF1A-EPAS1, which leads to the transcriptional upregulation of genes associated with the tumorigenic hypoxia response. One such gene is *VEGFA*, which can promote angiogenesis and the development of new blood vessels. As a result, elevated levels of HIF1A-EPAS1 play a crucial role in orchestrating angiogenesis in ccRCC. Notably, in the context of ccRCC growth, it is noteworthy that HIF1A and EPAS1 have contrasting impacts. Multiple studies reported that *HIF1A* may function as a tumor suppressor gene, whereas *EPAS1* acts as an oncogene [24,25,42]. EPAS1 is positioned upstream of many crucial neoplastic pathways and is widely regarded as a very promising target for therapeutic interventions in ccRCC [24,25,40,42]. As the above results indicate, ATL-I can inhibit ccRCC proliferation, invasion, and migration and exhibits robust antiangiogenic capacity by inhibiting the EPAS1-VEGFA signaling pathway. As expected, due to the significant role of ATL-I in regulating EPAS1 expression, ATL-I exhibited distinct selective cytotoxic effects on ccRCC cells compared with renal tubular epithelial cells.

Autophagy is a conserved catabolic degradation mechanism within cells in which long-lived proteins, protein aggregates, and damaged organelles are transported to autophagosomes that later fuse with lysosomes for degradation [29,43,44]. Autophagy has dual functions in the modulation of cancer. Autophagy plays a crucial role in preserving genomic stability and facilitating oncogene-induced senescence in the early stages of tumor development, thus impeding the progression toward malignancy. Nevertheless, in advanced stages, autophagy has the potential to aid the survival of tumor cells under stress conditions [45]. Although it is well known that EPAS1 can be degraded by the ubiquitin-proteasome system, recent studies have reported that there is a compensatory interaction between the ubiquitin-proteasome and autophagy-lysosome systems in EPAS1 degradation [29,33,34]. In the present study, ATL-I was identified as a late-stage autophagy agonist that directly degrades EPAS1 to suppress ccRCC tumorigenesis. Intriguingly, we did not find that ATL-I inhibited HIF1A, probably because HIF1A is mainly degraded by chaperone-mediated autophagy degradation rather than macroautophagy [46,47].

Autophagosome degradation is intricately connected to autolysosome formation and lysosomal function. The acidic environment of the lysosome is mainly attributed to the proton pump activity of V-ATPase [48]. The V-ATPase consists of a peripheral domain (V_1_) and an integral domain (V_0_) that translocate protons through the operation of a rotary mechanism [49]. ATP6V0D2 is a constituent of the V-ATPase complex, which assumes a critical function in mediating the interaction between the V_1_ and V_0_ domains [49]. In this study, we found that ATP6V0D2 was expressed at lower levels in ccRCC and regulated by ATL-I. Using multiple genetic approaches and in vitro and in vivo experiments, we found that the expression of ATP6V0D2 can be induced by ATL-I and promote the autophagy pathway. To uncover the underlying mechanism of ATP6V0D2 in autophagy promotion, we evaluated the function of ATP6V0D2 in various phases of the autophagy process. First, we observed that ATP6V0D2 can affect lysosomal acidification and activity and that *ATP6V0D2* knockdown could reverse these effects after ATL-I treatment. Interestingly, Murase *et al*. also reported that ATP6V0D2 plays a central role in the acidification of intracellular vesicles in macrophages [32]. However, a previous study reported that ATP6V0D1, not ATP6V0D2, was required for lysosome acidification and maturation in mouse macrophages [33]. This discrepancy most likely arises because ATP6V0D2 has diverse functions at different stages of cell differentiation or between species.

Next, we further investigated whether ATP6V0D2 contributes to the progression of autophagosome-lysosome fusion. Confocal colocalization analysis suggested that ATL-I promoted autophagosome and lysosome fusion through upregulation of ATP6V0D2, and knockdown of *ATP6V0D2* blocked this process. Actually, a recent study reported that ATP6V0D2 coordinated the final steps in autophagy by promoting the fusion of autophagosomes with lysosomes in macrophages [33]. Mechanistically, ATP6V0D2 binds to STX17 and VAMP8, promoting SNARE protein-mediated autophagosome-lysosome fusion, which was also validated in ccRCC cell lines. In addition, one superior finding of our work is that ATP6V0D2 directly binds to RAB7 and VPS41 and acts as an important bridge to promote the RAB7-HOPS interaction. Therefore, we propose a potential mechanism by which ATP6V0D2 promotes autophagy in ccRCC. On the one hand, ATP6V0D2 promotes the RAB7-HOPS interaction and facilitates SNARE complex assembly, contributing to autophagosome – lysosome fusion. On the other hand, ATP6V0D2 promotes autolysosome degradation by increasing the acidification and activity of lysosomes.

TKIs have been extensively used as treatment agents for ccRCC. However, the effectiveness of these treatments is constrained by the presence of resistance mechanisms. Recent research has shown that the elevated resistance of RCC to TKIs may be attributed to the overexpression of members of the VEGFA pathway, which serves as a compensatory mechanism in response to diminished KDR signaling upstream [10,11,26,50–52]. Our study revealed a significant upregulation of EPAS1 in ccRCC cells that exhibited resistance to sunitinib treatment. Moreover, this upregulation of EPAS1 was shown to be correlated with an unfavorable prognosis for ccRCC patients who had sunitinib therapy [52]. Considering that ATL-I induced ATP6V0D2 upregulation, which regulated the EPAS1-VEGFA pathway. We hypothesized that ATL-I might serve as a potential medication for ccRCC patients resistant to TKIs. As expected, the combination of ATL-I with sunitinib partially restored sensitivity to sunitinib in SU-R-786O cells.

In summary, our study has shown that ATL-I possesses the capability to target various cancer-related pathways, thereby impeding the progression of ccRCC. In particular, anti-angiogenesis represents a crucial mechanism by which ATL-1 exert its profound impact, achieved through suppressing the EPAS1-VEGFA signaling axis. Mechanistically, ATL-I promoted the autophagic degradation of EPAS1 by upregulating ATP6V0D2 to increase lysosomal activity and promote the fusion of autophagosomes and lysosomes. Additionally, we found that hyperactivation of EPAS1 was a possible mechanism of sunitinib resistance in ccRCC, and ATL-I partially reversed this resistance. Therefore, our study supports ATL-I as a potential antitumor lead compound for ccRCC treatment.

## Materials and methods

### Cell lines and tissue samples

The cell lines HK2, ACHN, 786O, and OSRC2 were procured from the American Type Culture Collection and Procell Life (CRL-2190, CRL-1611, CRL-1932; CL-0177). The sunitinib-resistant 786O cell line (Su-R-786O) was established by exposing 786O cells to an initial dose of sunitinib (2 µM; MedChemExpress, HY-10255A) and gradually increasing the concentration of sunitinib up to 14 µM for 16 weeks, followed by culture media containing 10 µM sunitinib. Human umbilical vein endothelial cells (HUVECs) were sourced from ScienCell (8000) and maintained according to the guidelines provided by the manufacturer. All cell lines were authenticated as per standard protocols and grown in a sterile incubator environment set at 37°C with a 5% CO_2_. To induce hypoxia, the cells were placed in a specialized hypoxia chamber (Thermo Fisher Scientific) that maintained an oxygen concentration of 0.1%.

The study enrolled patients diagnosed with ccRCC who had undergone partial or radical nephrectomy at either Tongji Hospital or the First Affiliated Hospital of Shihezi University between July 2020 and March 2023. Ethical approval for the study was obtained from the Tongji Hospital Ethics Committee (TJ-IRB-20230331) and the Ethics Committee of the First Affiliated Hospital of Shihezi University (KJ2023-056-01). All participants provided their informed consent, agreeing to the utilization of the tissue samples for scientific research.

### Chemical reagents

Cells were treated with bevacizumab (MedChemExpress, HYP9906) at 50 ng/ml or ATL-I (MedChemExpress, HY-N0201) at specified concentrations. Cycloheximide (MedChemExpress, HY-12320), an inhibitor of protein synthesis, was used to test whether ATL-I or ATP6V0D2 affects the protein degradation rate of EPAS1. The effects of ATP6V0D2 on EPAS1 protein expression via proteasomal or lysosomal degradation pathways were investigated using proteasome inhibitor MG132 (Beyotime Biotechnology, S1748) and lysosomal inhibitor chloroquine (MedChemExpress, HY17589A). Autophagy was induced with 50 nM rapamycin (MedChemExpress, HY10219) for 4–12 h.

### Plasmids and cell transfection

To knock down *ATP6V0D2* expression, short hairpin RNA (sh-*ATP6V0D2*) and non-target shRNA (sh-nc) were developed and synthesized by Genomeditech with the following sequences: sh-1#: 5′-ATATCTTCATGAGTTGCAAAT-3′; sh-2#: 5′-CCAGACTACTGATATGGTAA-3′; and sh-3#: 5′-GCAGAATGTATCACAGA-3′. sh-*ATG5* and sh-nc were also designed and synthesized by Genomeditech with the following sequences: sh-1#: 5′-GCAACTCTGGATGGGATTGTT-3′; sh-2#: 5′-GGAATATCCTGCAGAAGAATT-3′; and sh-3#: 5′-ACTTACTACGGATATTGTAA-3′. Flag-tagged ATP6V0D2 (GeneID: 245972; vector: pGMLV) was purchased from Genomeditech. GST-tagged ATP6V0D2 (Gene ID: 245972; vector: pGEX-4T-1), His-tagged RAB7 (Gene ID: 7879; vector: pGMLV), and His-tagged VPS41 (Gene ID: 27072; vector: pGMLV) were purchased from Tsingke Biotechnology. Using RT-qPCR and western blot, the transfection efficiency was validated. Following transduction, the cells were subjected to puromycin selection to establish stable cell lines for subsequent experiments.

### CCK-8, EdU, and colony formation assays

Cells were inoculated into 96-well plates at a concentration of 2000 cells per well, utilizing a medium supplemented with 10% FBS. The assessment of cell viability was conducted in accordance with the guidelines of the CCK-8 protocol (Yeasen Biotechnology, 40203ES60). To execute the EdU assay, cells were grown in 12-well plates at a seeding density of 8 × 10^4^ cells per well. Following exposure to various experimental conditions, the cells were incubated with a medium enriched with 50 μM EdU for a duration of 2 h. The cells were subjected to fixation for 20 min at ambient temperature using a 4% paraformaldehyde solution. Subsequently, the cells were stained with Hoechst reagent and Apollo staining solution (RiboBio, C10310). A fluorescence microscope was used to assess the proportion of EdU-positive cells in five randomly selected fields (Olympus IX71, Tokyo, Japan). Moreover, HK-2 cells were seeded at a density of 600 cells per well in 6-well plates and were treated with either ATL-I or DMSO. After 14 days, the colonies were counted and statistically analyzed.

### Cell migration and invasion assays

The migratory and invasive capabilities of the cells were evaluated through wound healing and Matrigel invasion assays. For cell migration, cells were plated in 6-well plates at a seeding density of 2 × 10^5^ cells per well. Upon achieving a confluence of approximately 90%, a standardized wound was introduced by gently scraping the cell monolayer with a 1-mL pipette tip. The rate of wound closure was quantified after a 24-h interval using the ImageJ software. For the invasion assay, the transwell chamber system was utilized. The top chamber of the transwell was coated with Matrigel (ABW Matrigengel 8,027,045) for 30 min at 37°C. Cells were then plated in the top chamber at a concentration of 3 × 10^4^ cells per well in a serum-free medium, while the bottom chamber was supplemented with 500 µL of medium enriched with 10% FBS. After a 24-h incubation at 37°C in an environment maintained at 5% CO_2_, cells that had penetrated the upper membrane surface were immobilized using a 4% paraformaldehyde solution. Subsequently, these cells were subjected to a staining process using a 0.1% crystal violet solution for 60 min. The cells in five randomly selected fields were enumerated using a microscope.

### Tube formation assay

Matrigel was plated evenly at 200 μl/well in a 48-well plate and incubated at 37°C for 40 min to polymerize. HUVECs pretreated with DMSO or ATL-I for 36 h were harvested, plated onto precoated Matrigel at a density of 3 × 10^6^ cells per well and incubated at 37°C for 2.5 h. The target cells were labeled with 2 μM calcein-AM (Beyotime Biotechnology, C2012) for 30 min at 37°C and subsequently imaged via fluorescence microscopy.

### Chick chorioallantoic membrane

The antiangiogenic capacity of ATL-I was assessed using the CAM assay. Specific pathogen-free fertilized chicken eggs were obtained from the Boehringer Ingelheim Biology Company. The fertilized eggs were incubated at 38.5°C with 5% CO_2_ and 50% humidity for 15 days. Then, a small window (approximately 2 cm in diameter) was created on the eggshell in a sterile environment to expose the CAM. The sterile Teflon O-ring (Chemours, AF 1600 X) was gently placed on the CAM, and the drug was added inside the ring. The window was sealed with adhesive tape, and the eggs were returned to the incubator. After 72 h, the number of blood vessels was manually quantified under a stereomicroscope (Leica M165 C, Germany).

### Flow cytometry analysis of cell apoptosis and the cell cycle

To ascertain the apoptotic rate among the cells, they were initially harvested and subjected to a double wash with PBS (Boster Biological Technology, PYG0021). Following this, the cells underwent a dual-staining protocol using propidium iodide and ANXA5/annexin V (Yeasen Biotechnology, 40303ES20). The staining procedure was conducted in a dark chamber at ambient temperature for a duration of 10 to 15 min to allow for adequate binding. For the assessment of cell cycle distribution, the cells were first fixed with a 70% ethanol solution and stored at 4°C overnight. This fixation step was followed by an incubation period with RNase and propidium iodide (Yeasen Biotechnology, 40301ES50) for 30 min. The acquisition of flow cytometry data was facilitated by utilizing a CytoFlex cytometer (Beckman Coulter, USA). The subsequent data analysis was meticulously conducted employing FlowJo V10 software.

### Lysosomal acidification and activity assays

Lysosomal acidification assay was performed according to previous methods according to a detailed protocol [53,54]. Briefly, the cell culture medium was replaced with prewarmed working solution containing 2 μM LysoSensor Green DND-189 (Yeasen Biotechnology, 40767ES), and incubated for 30 min at 37°C. For the lysosomal activity assay, the LysoTracker^TM^ Deep Red probe (Thermo Fisher Scientific, L12492) was used according to the manufacturer’s instructions. Briefly, the cells were incubated with LysoTracker^TM^ working solution (10 nM) at 37°C for 30 min in the dark. Then, the cells were washed with PBS and quantitatively evaluated via flow cytometry (Beckman Coulter, USA).

### Rt-qPCR and rna-seq

The process of extracting total cellular RNA was executed utilizing the TRIzol reagent (Servicebio Technology, G3013). The synthesis of the first-strand cDNA was facilitated by the Hifair II 1st Strand cDNA Synthesis Kit (Yeasen Biotechnology, 11119WS). qPCR analysis was carried out following the SYBR Green Master Mix protocol, using a step one PCR instrument (Applied Biosystems, Foster, CA, USA). *GAPDH* served as the endogenous reference gene for normalizing the expression levels of the mRNA. The sequences of the primers employed in the qPCR reactions are detailed in Table S1. To elucidate the alterations in the transcriptome in response to ATL-I treatment, sequencing libraries were prepared from the total RNA isolated from both the control and ATL-I treated groups, each consisting of three biological replicates. Subsequently, these libraries were subjected to next generation sequencing to reveal the transcriptomic landscape.

### Western blot analysis

Protein extraction from cells was achieved using a RIPA buffer (Boster Biological Technology, AR0102), which was enriched with a cocktail of protease and phosphatase inhibitors (Boster Biological Technology, AR1193 and AR1183). The concentration of the extracted proteins was determined by employing a BCA protein assay kit (Boster Biological Technology, AR1189). The protein extracts were subsequently separated via SDS-PAGE and subsequently transferred onto a polyvinylidene difluoride membrane (Bio-Rad Corporation 1,620,256). The membranes were sectioned according to the molecular weights of the target proteins. Following this, the membranes were subjected to incubation with primary and secondary antibodies. The visualization of the protein bands was achieved using the P Pierce ECL substrate WB detection system (Bio-Rad Laboratories 12,003,154). The specific primary and secondary antibodies utilized in this study are detailed in Table 1.

**Table 1.** Primary and secondary antibodies used in the study.

Antibody	Company (Cat. No.)	Working dilutions
Primary Antibody		
ATP6V0D2	Abmart (PH8735)	WB 1:2000; IHC 1:500
ATG5	Cell Signaling Technology (CST; 12994S)	WB 1:1000
HIF1A	Affinity (BF8002)	WB 1:1000
EPAS1/HIF2α	CST (71565S)	WB 1:1000; IF 1:100
EPAS1/HIF2α	Abcam (ab109616)	IHC 1:100
VEGFA	Proteintech (19003-1-AP)	WB 1:1000; IHC 1:100
PECAM1/CD31	Abcam (ab28364)	IHC 1:50
TJP1/ZO-1	Abcam (ab190085)	WB 1:1000
CDH2/N-cadherin	Abcam (ab98952)	WB 1:1000
VIM/vimentin	Abcam (ab8978)	WB 1:1000
MTOR	CST (2983)	WB 1:1000
PI3K	CST (4249)	WB 1:1000
STX17	Proteintech (17815-1-AP)	WB 1:1000; IF 1:100
VAMP8	Abcam (ab76021)	WB 1:10000; IF 1:250
p-AKT	CST (4060P)	WB 1:1000
AKT	CST (9272S)	WB 1:1000
MAPK	Affinity (AF6456)	WB 1:1000
CASP3	Abcam (ab32351)	WB 1:5000
c-CASP3	Abcam (ab32042)	WB 1:500
BCL2	Abcam (ab32124)	WB 1:1000
BAX	Abcam (ab32503)	WB 1:5000
SQSTM1/p62	Abcam (ab240635)	WB 1:1000
LC3A/B	CST (4108S)	WB 1:1000; IF 1:100
LAMP1	Abcam (ab289548)	WB 1:1000; IF 1:100
CTSB	Proteintech (12216-1-AP)	WB 1:2000
CTSD	Proteintech (21327-1-AP)	WB 1:5000
RAB7	CST (9637S)	WB 1:1000; IF 1:100
VPS39	Proteintech (16219-1-AP)	WB 1:800
VPS41	Proteintech (13869-1-AP)	WB 1:800
VPS41	Thermo Fisher (PA5 -100,366)	IF 1:200
MKI67/Ki-67	Abcam (ab16667)	IHC 1:200
Mouse anti-Flag	Abclone (AE005)	WB 1:2000
Rabbit anti-Flag	Abclone (AE063)	WB 1:2000
Mouse anti-His	Abclone (AE003)	WB 1:2000
Rabbit anti-GST	Abclone (AE006)	WB 1:1500
GAPDH	Proteintech (60004-1-Ig)	WB 1:5000
Secondary Antibody		
Goat Anti-Rabbit	Abclone (AS014)	WB 1:5000
Goat Anti-Mouse	Abclone (AS003)	WB 1:5000
Cy3 Goat Anti-Rabbit	Servicebio (GB21303)	IF 1:500
Cy3 Donkey Anti-Mouse	Servicebio (GB21401)	IF 1:500
FITC Goat Anti-Mouse	Servicebio (GB22301)	IF 1:500
FITC Goat Anti-Rabbit	Servicebio (GB22303)	IF 1:500

### Mass spectrometry, coimmunoprecipitation, and GST affinity-isolation assays

Mass spectrometry analysis of proteins pulled down by FLAG-tagged ATP6V0D2 was performed by SpecAlly Biology Company (Q Exactive HF, Thermo Fisher Scientific, USA). Coimmunoprecipitation assays were performed according to the instructions of Biolinkedin Co-IP (Biolinkedin, IK1004). The treated cells were lysed on ice for 30 min in cell lysis buffer containing 1% protease inhibitor and 1% phosphatase inhibitor. The cell lysates and antibodies were subjected to overnight incubation at a temperature of 4°C with rotational agitation, resulting in the formation of immunological complexes. Precooled magnetic beads were added to the immune complexes, which were subsequently incubated for 2 h at room temperature. The beads were washed 3 times with wash buffer, collected, added to SDS-PAGE sample loading buffer, boiled to obtain antigen-antibody complexes bound to the beads, and subsequently subjected to western blot.

The recombinant GST-ATP6V0D2, His-RAB7, and His-VPS41 proteins were expressed in BL21 (Rosetta) bacterial cells and purified with glutathione – Sepharose beads (Biolinkedin, PK2004) and His beads (Biolinkedin, PK2005), respectively, according to the instructions provided for the kit. The GST affinity-isolation assays were conducted with the GST Protein Interaction Pull-Down Kit (Biolinkedin, IK2004) in accordance with the instructions provided by the manufacturer. Briefly, we immobilized pure GST-tagged proteins (2.5 μg) onto glutathione-Sepharose beads. These beads were then incubated with His-RAB7, His-VPS41, or a combination of both in affinity-isolation buffer supplemented with PMSF (Boster Biological Technology, AR1192). Following the incubation period, the mixture containing the protein-magnetic bead complexes was carefully transferred to a magnetic tube rack, enabling the separation of the magnetic beads with bound proteins. The isolated protein-enriched beads were then prepared for further analysis via western blot.

### Immunofluorescence staining and TEM

Cell samples subjected to the experimental treatment were seeded onto glass coverslips within 12-well culture plates at a concentration of 15 × 10^4^ cells per well. Following a rinse with PBS, the cells were fixed using a 4% paraformaldehyde solution for a duration of 30 min under ambient conditions. Post-fixation, the cells underwent permeabilization with a 0.5% Triton X-100 (Solarbio, T8200) solution for 15 min, after which they were rinsed again with PBS. Subsequent to a blocking step with goat serum (Solarbio, SL038), the cells were incubated with the primary antibody overnight at 4°C. This was succeeded by a staining procedure with fluorescently labeled secondary antibodies. The coverslips were then thoroughly washed twice with PBS and subsequently stained with DAPI for a period of 10 min. For the LysoTracker Deep Red (Thermo Fisher Scientific, L12492), the cells were incubated with LysoTracker working solution (10 nM) at 37°C for 30 min in the dark prior to paraformaldehyde fixation. Images were obtained using a confocal microscope (LSM880, ZEISS, Germany). Details of the primary and secondary antibodies utilized are provided in Table 1.

For the TEM experiment, treated 786O cells were washed three times in PBS and fixed with 2.5% glutaraldehyde in phosphate-buffered saline (pH 7.4) and 1% (v:v) osmium tetroxide for 2 h. The samples were then dyed with 2% uranyl acetate for 30 min, washed three times with distilled water and dehydrated for 15 min. Subsequently, the samples were embedded in EPON 812 resin (Head Biotechnology, E8000) and cured for 24 h at 70°C. Ultrathin slices were procured using an ultramicrotome and then subjected to staining with a solution containing 2% uranyl acetate and lead citrate. The samples were imaged with a transmission electron microscope (HT7800, Hitachi Company, Japan).

### Animal studies

The Institutional Animal Care and Use Committee of Huazhong University of Science and Technology (TJH201910012) granted approval for all animal experiments conducted in this study. The female athymic nude mice used in this study were obtained from Shulaibao Biotechnology at 5 weeks of age. Angiogenesis was evaluated using a Matrigel plug assay. 786O cells (1 × 10^6^) were mixed with ATL-I or DMSO and resuspended at a ratio of 1:2 with Matrigel in 200 µl. The mixture was injected subcutaneously into the back of nude mice. After 14 days, the vessels of the transplanted tumors were counted, and the tumors were removed for immunohistochemical staining for PECAM1 and VEGFA. To establish the subcutaneous tumor xenograft model, 2 × 10^6^ cells were injected subcutaneously into the mice. A 7-day interval was allowed for tumor initiation and growth, followed by random distribution of the mice into distinct groups. Tumor size were measured every 4 days. After 28 days, the mice were humanely euthanized, and the tumors were excised, measured, and preserved for further investigation. For construction of the orthotopic xenograft model of ccRCC, 1 × 10^6^ 786O cells resuspended in 100 μL of fresh growth medium were injected into the left kidneys of the mice as described previously [55]. After 4 weeks of treatment with ATL-I (50 mg/kg), the mice were euthanized, and the tumors were removed and stored for additional analysis. In the experiment addressing ATL-I reversal of sunitinib resistance, 2 × 10^6^ 786O or Su-R-786O cells were injected subcutaneously into mice. The mice were then randomly allocated into five groups. The mice in these groups were subjected to daily intraperitoneal injections of the indicated treatments. The experimental conditions were as follows: (1) WT-786O cells + DMSO, (2) WT-786O cells + sunitinib (20 mg/kg), (3) WT-786O + sunitinib (20 mg/kg) + ATL-I (50 mg/kg), (4) Su-R-786O + sunitinib (20 mg/kg) and (5) Su-R-786O + sunitinib (20 mg/kg) + ATL-I (50 mg/kg). The tumor volumes were measured at regular intervals of 4 days. After a 28-day treatment period, the mice were euthanized, and the tumors were extracted for further examination.

### Bioinformatics analysis

The expression and clinical data were sourced from the comprehensive repository of TCGA. A comparative analysis was performed to evaluate the differential expression levels of the *ATP6V0D2* gene in malignant tissues and para-cancerous tissues, utilizing the Wilcoxon test. This analysis was supplemented by an investigation into the potential correlations between *ATP6V0D2* expression levels and a spectrum of clinicopathological features characteristic of ccRCC. To elucidate the prognostic significance of *ATP6V0D2*, Kaplan-Meier survival curves were employed to assess the relationship between *ATP6V0D2* expression and key survival endpoints in ccRCC patients, specifically overall survival, progression-free survival, and disease-specific survival.

### Statistical analysis

The results are reported as the arithmetic means accompanied by their standard deviations. For the comparative analysis of two groups, we employed Student’s t-test for data sets exhibiting normal distribution and the Wilcoxon test for those with non-normal or skewed distributions. The survival data were analyzed using the log-rank test to assess the statistical significance of differences in survival curves. All statistical computations were performed using R version 4.1.0 and GraphPad Prism version 7. A p-value threshold of less than 0.05 was set to denote a statistically significant result.

## Supplementary Material

Supplementary Material.docx

## Data Availability

All the data can be obtained by contacting the corresponding author.
