# Supplementary material for: Atractylenolide I inhibits angiogenesis and reverses sunitinib resistance in clear cell renal cell carcinoma through ATP6V0D2-mediated autophagic degradation of EPAS1/HIF2α
Source: Autophagy. 2024 Oct 30;21(3):619–38. doi: 10.1080/15548627.2024.2421699 (PMC11849937; doi:10.1080/15548627.2024.2421699)
Supplement: Supplementary Material.docx [file KAUP_A_2421699_SM3278.docx]

**Supplementary Material**

**Atractylenolide I inhibits angiogenesis and reverses sunitinib resistance in clear cell renal cell carcinoma through ATP6V0D2-mediated autophagic degradation of EPAS1/HIF2α**

*Qinyu Li^1,^ †, Kai Zeng^1, 2,^ †, Qian Chen^3, 4,^ †, Chenglin Han^1^, Xi Wang^5^, Beining Li^1^, Jianping Miao^6^, Bolong Zheng^7^, Jihong Liu^1^, Xianglin Yuan^5, *^, Bo Liu ^5, *^*

^1^Department of Urology, Tongji Hospital, Tongji Medical College, Huazhong University of Science and Technology, Wuhan, Hubei, China

^2^Department of Urology, the First Affiliated Hospital of Shihezi University, Shihezi, Xinjiang, China

^3^Hepatic Surgery Center, Tongji Hospital, Tongji Medical College, Huazhong University of Science and Technology, Wuhan, Hubei, China.

^4^Department of Hepatobiliary Surgery, The First Affiliated Hospital of Shihezi University, Shihezi, Xinjiang, China.

^5^Department of Oncology, Tongji Hospital, Tongji Medical College, Huazhong University of Science and Technology, Wuhan, Hubei, China

^6^Department of Geriatrics, Tongji Hospital, Tongji Medical College, Huazhong University of Science and Technology, Wuhan, Hubei, China

^7^School of Computer Science and Technology, Huazhong University of Science and Technology

† These authors contributed to this work equally and shared first authorship.

**Corresponding to:**

**Professor Bo Liu**, [boliu888@hotmail.com](mailto:boliu888@hotmail.com)

**Professor Xianglin Yuan**, [yuanxianglin@hust.edu.cn](mailto:yuanxianglin@hust.edu.cn)

**Supplementary Figures**

**
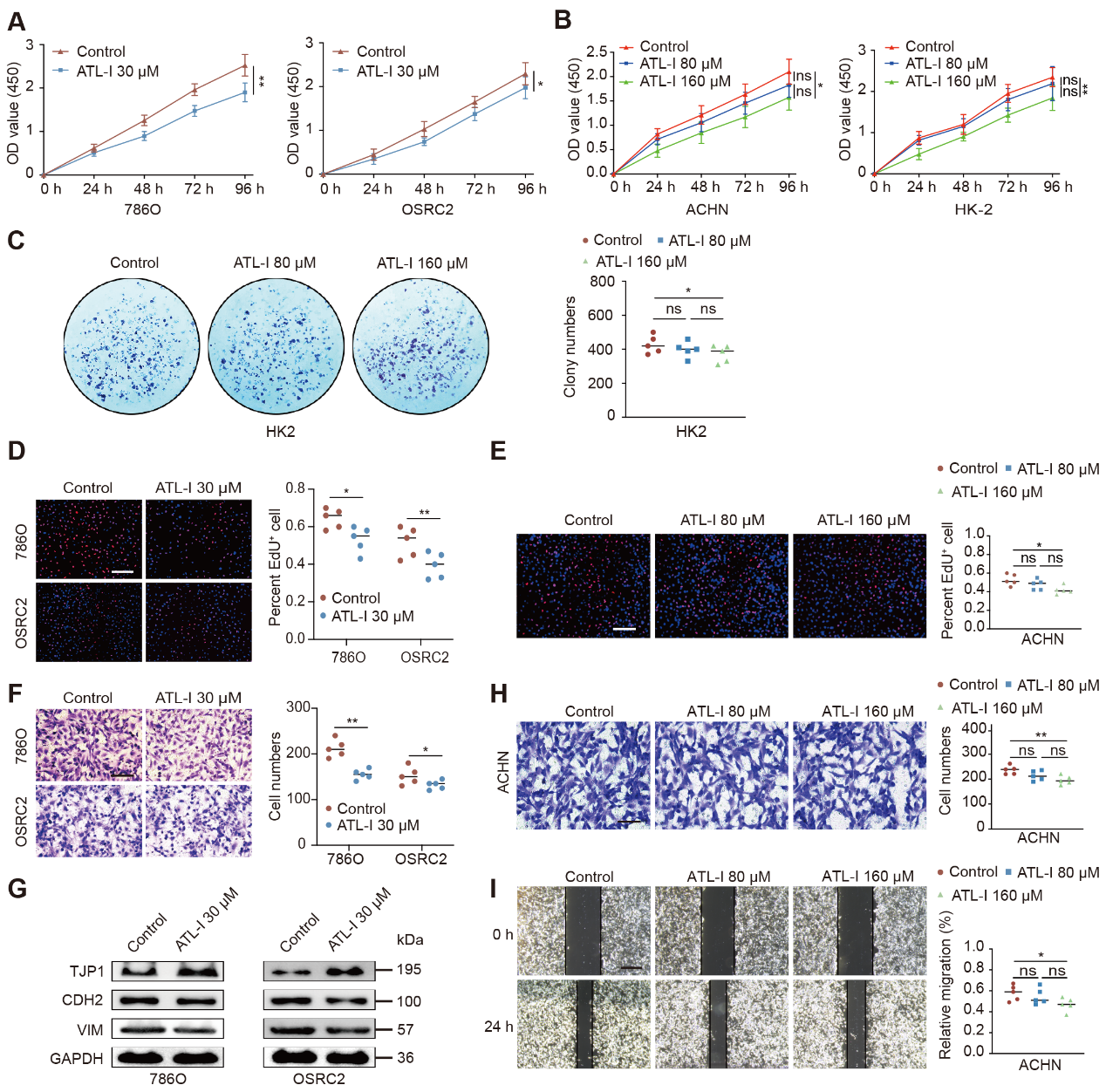
Figure S1.** ATL-I inhibits RCC proliferation, invasion, and migration. (**A**) The impact of ATL-I at a concentration of 30 μM on the viability of 786O and OSRC2 cells was assessed over a period ranging from 0 to 96 h. (**B**) Effect of ATL-I on ACHN and HK-2 cell viability at different time points (0 to 96 h). (**C**) Colony formation assays were performed to evaluate HK2 cell proliferation after incubation with 0, 80, or 160 μM ATL-I. (**D**) Cell proliferation post-exposure to 0 or 30 μM ATL-I for 48 h was quantified using the EdU assay. Scale bar: 100 μm. (**E**) After treatment with 0, 80, or 160 μM ATL-I for 48 h, ACHN cell proliferation was determined by EdU assay. Scale bar: 100 μm. (**F**) Invasion capabilities were evaluated through Transwell assays following a 48-h incubation with 0 or 30 μM ATL-I. Scale bar: 50 μm. (**G**) Expression levels of EMT markers were compared between control and treatment groups administered 30 μM ATL-I. (**H and I**) Transwell and wound healing assays were performed to evaluate ACHN cell invasion and migration after incubation with 0, 80, or 160 μM ATL-I. Scale bar: 50 or 100 μm. (* p < 0.05, ** p < 0.01.)

**
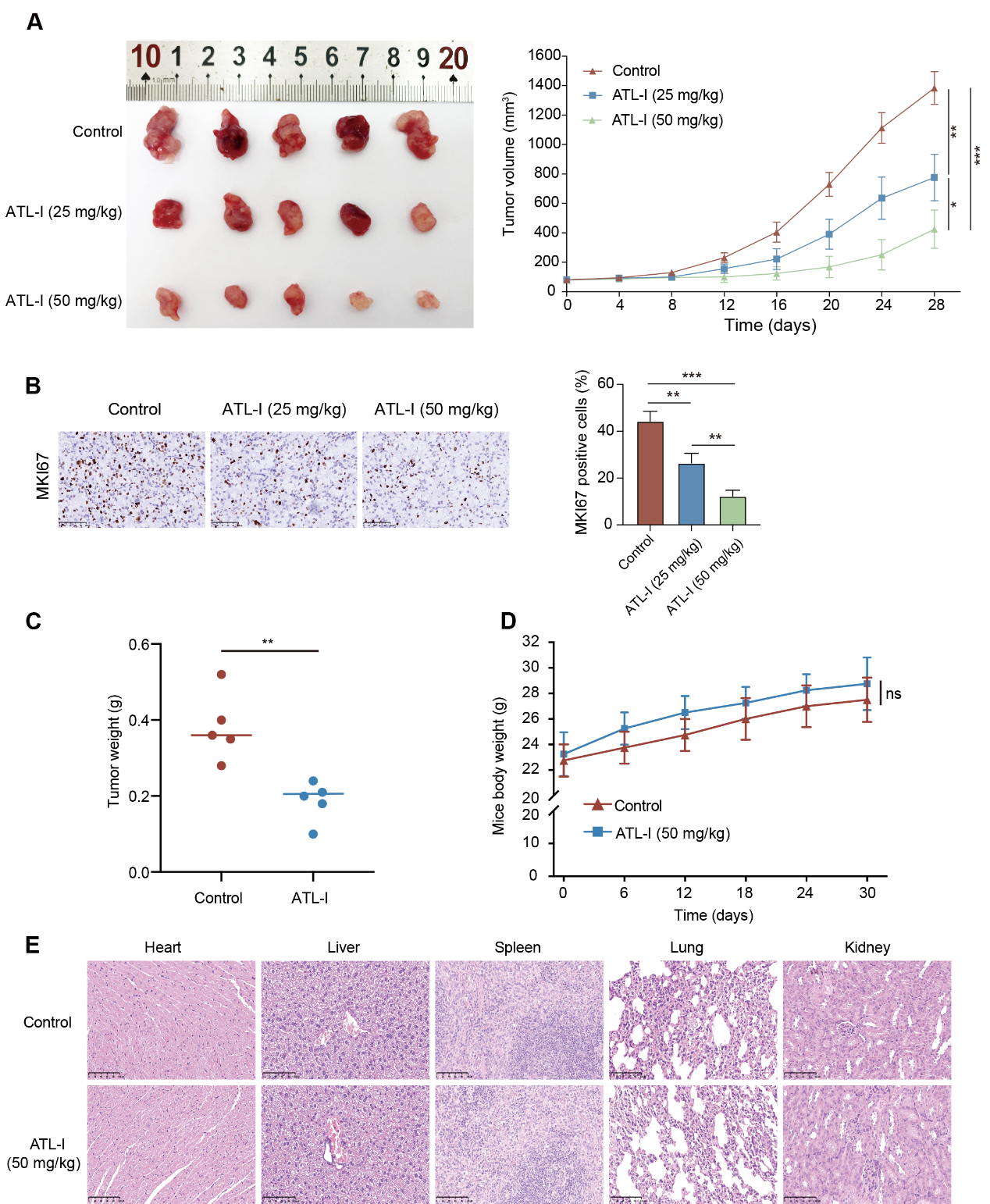
Figure S2.** ATL-I suppresses the proliferation of ccRCC in vivo. (**A**) The tumor growth curves of subcutaneous xenografts from the vector and ATL-I treatment groups (25 and 50 mg/kg). (**B**) Immunohistochemical analysis was used to detect the protein expression levels of MKI67 in each group. Scale bar: 100 μm. (**C**) Establishment of an orthotopic xenograft model using wildtype 786O cells, followed by tumor excision from the kidney, weighing, and statistical analysis. (**D**) Comparison of body weights between the two groups. (**E**) H&E staining was used to compare histomorphological changes in the heart, liver, spleen, lung, and kidney tissues between the two groups. Scale bar: 100 μm. (* p < 0.05, ** p < 0.01, *** p < 0.001.)

**
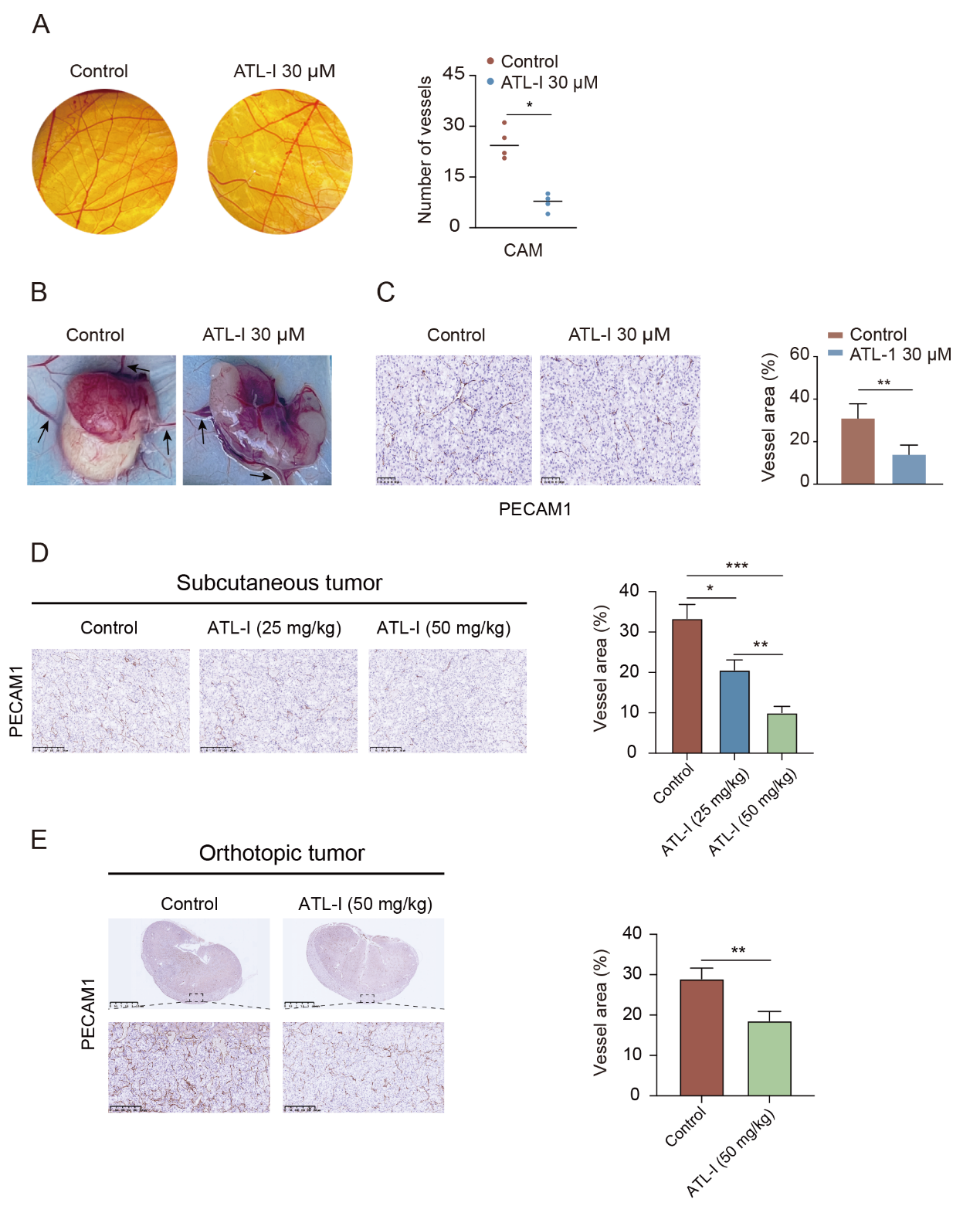
Figure S3.** ATL-I inhibits angiogenesis in vivo. (**A**) The inhibitory effect of 30 μM ATL-I on neo-vascularization was observed in the CAM assay. (**B**) Representative images of Matrigel plug assays following treatment with either DMSO or 30 μM ATL-I. (**C**) IHC staining for PECAM1 in both control and ATL-I-treated Matrigel plugs was conducted. The vascular area was then measured and the percentages were compared. Scale bar: 100 μm. (**D and E**) Immunohistochemical analysis was employed to ascertain the expression levels of PECAM1 in both subcutaneous (**D**) and orthotopic (**E**) tumor tissues, comparing the Control and ATL-I treatment groups. Scale bar: 2.5 mm or 100 μm. (* p < 0.05, ** p < 0.01, *** p < 0.001.)

**
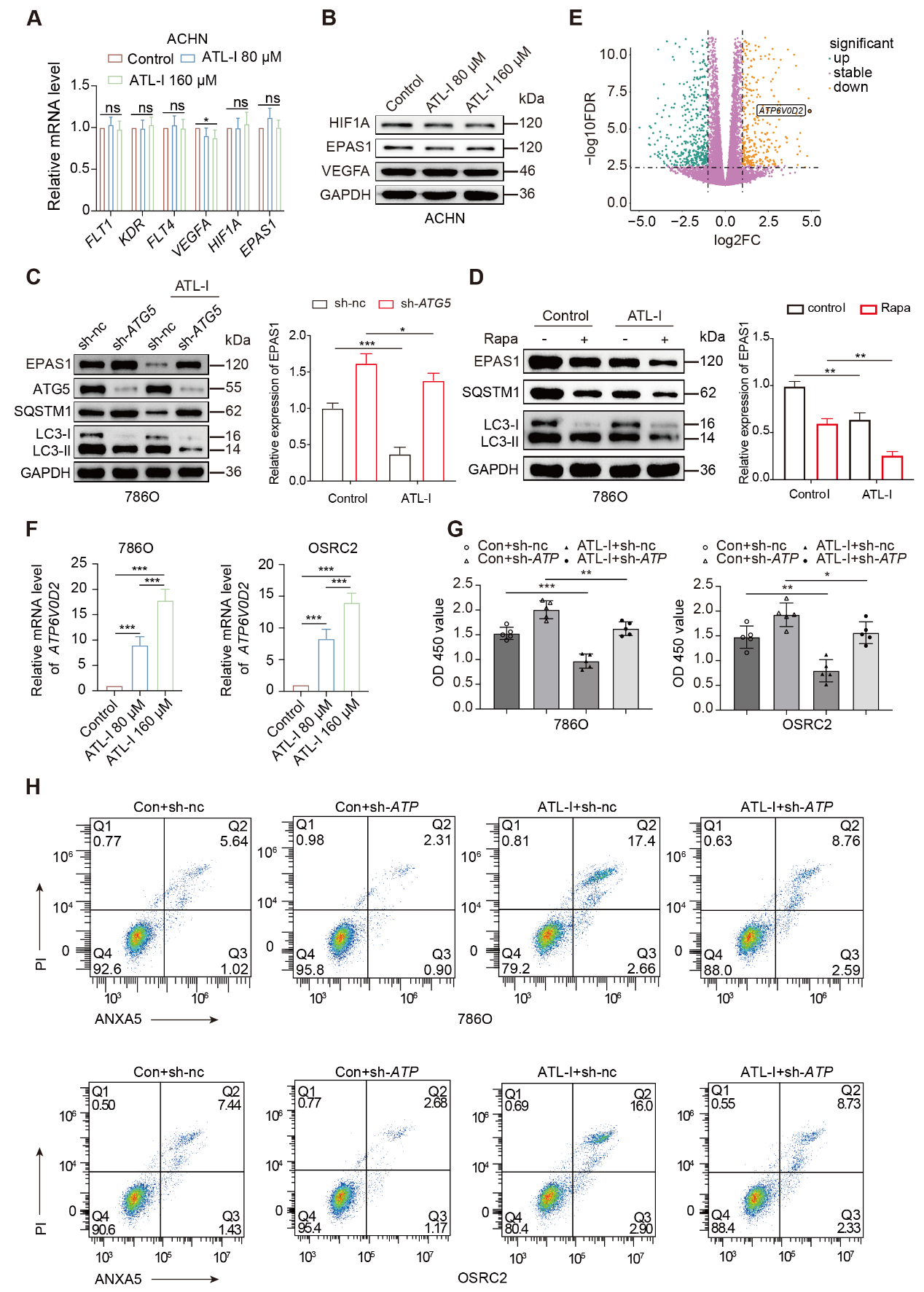
Figure S4.** Upregulation of ATP6V0D2 by ATL-I accelerated the autophagic degradation of EPAS1. (**A**) Expression analysis of *HIF1A*, *EPAS1*, *VEGFA*, *FLT1*, *KDR*, and *FLT4* by RT-qPCR in ACHN cells treated with 0, 80, or 160 μM ATL-I for 48 h. (**B**) Immunoblotting was performed to detect the protein levels of HIF1A, EPAS1 and VEGFA in ACHN cells treated with 0, 80, or 160 μM ATL-I for 72 h. (**C**) Control and *ATG5*-knockdown 786-O cells were treated with an equivalent dose of DMSO or 80 μM ATL-I for 36 h. Western blot analysis was performed to detect the expression levels of EPAS1, ATG5, SQSTM1, and LC3 in each group of cells. (**D**) 786O cells were exposed to rapamycin (50 nM) for 6 h, after which western blot analysis was performed to detect the expression levels of EPAS1, SQSTM1, and LC3. (**E**) Volcano plot representation of differential gene expression. (**F**) Expression analysis of *ATP6V0D2* by RT-qPCR in 786O and OSRC2 cells treated with 0, 80, or 160 μM ATL-I for 48 h. (**G and H**) In rescue experiments, cell viability and apoptosis were evaluated by CCK-8 and flow cytometry in the indicated 786O and OSRC2 cells. (* p < 0.05, ** p < 0.01, *** p < 0.001.)

**
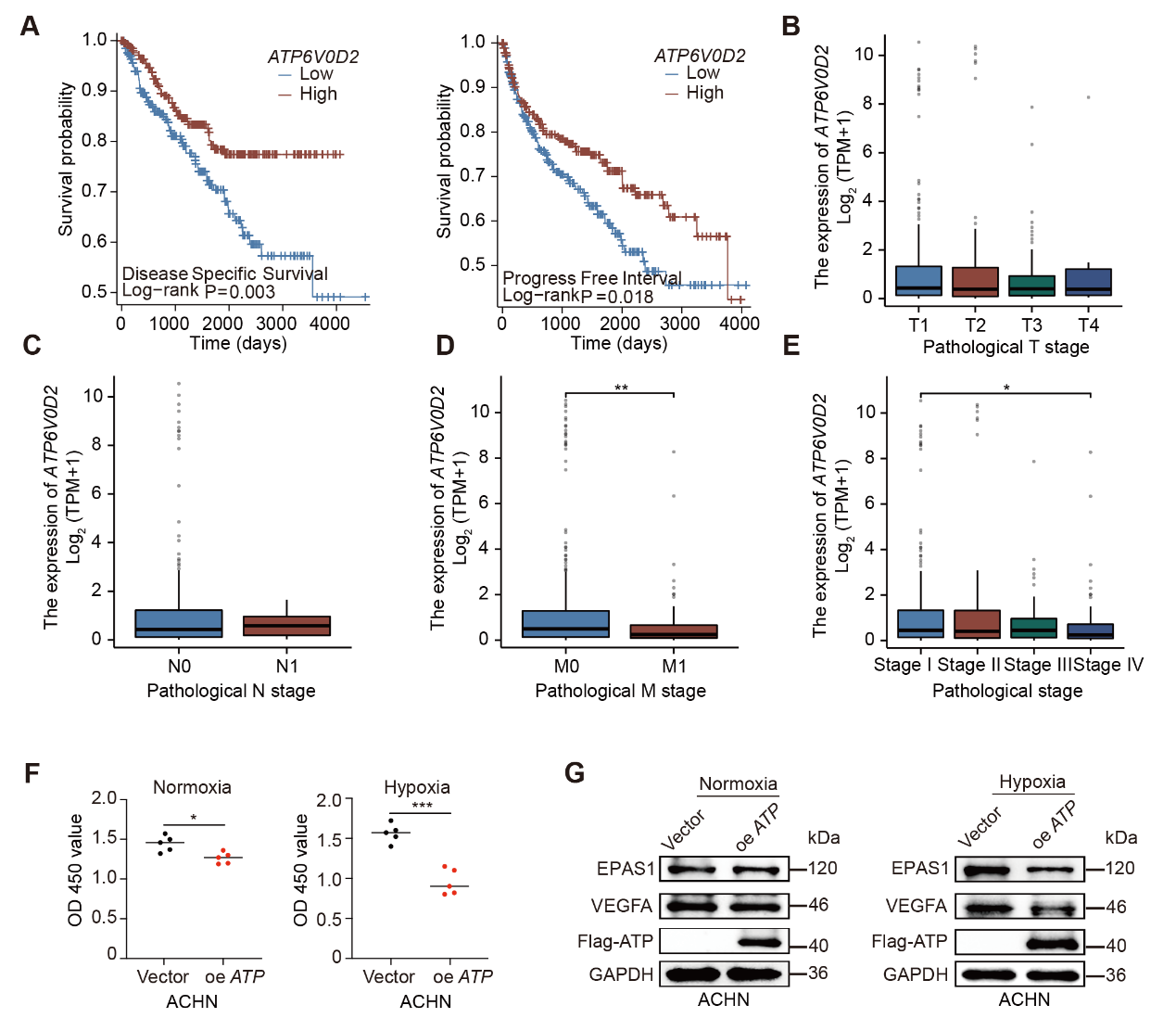
Figure S5.** ATP6V0D2 was negatively correlated with tumor progression and clinical outcome in RCC. (**A**) Differences in disease specific survival and progress free survival between *ATP6V0D2*^high^ and *ATP6V0D2*^low^ patients. (**B-E**) The expression of *ATP6V0D2* was related to various clinicopathological factors: T stage (**B**), lymph node metastasis (**C**), distant metastases (**D**), and pathological stage (**E**). (**F and G**) Cell viability and EPAS1 and VEGFA protein levels were assessed using the CCK8 assay and immunoblotting in vector and oe *ATP6V0D2* ACHN cells cultured under normoxic (**F**) or hypoxic (**G**) conditions for 24 h. (* p < 0.05, ** p < 0.01, *** p < 0.001.)

**
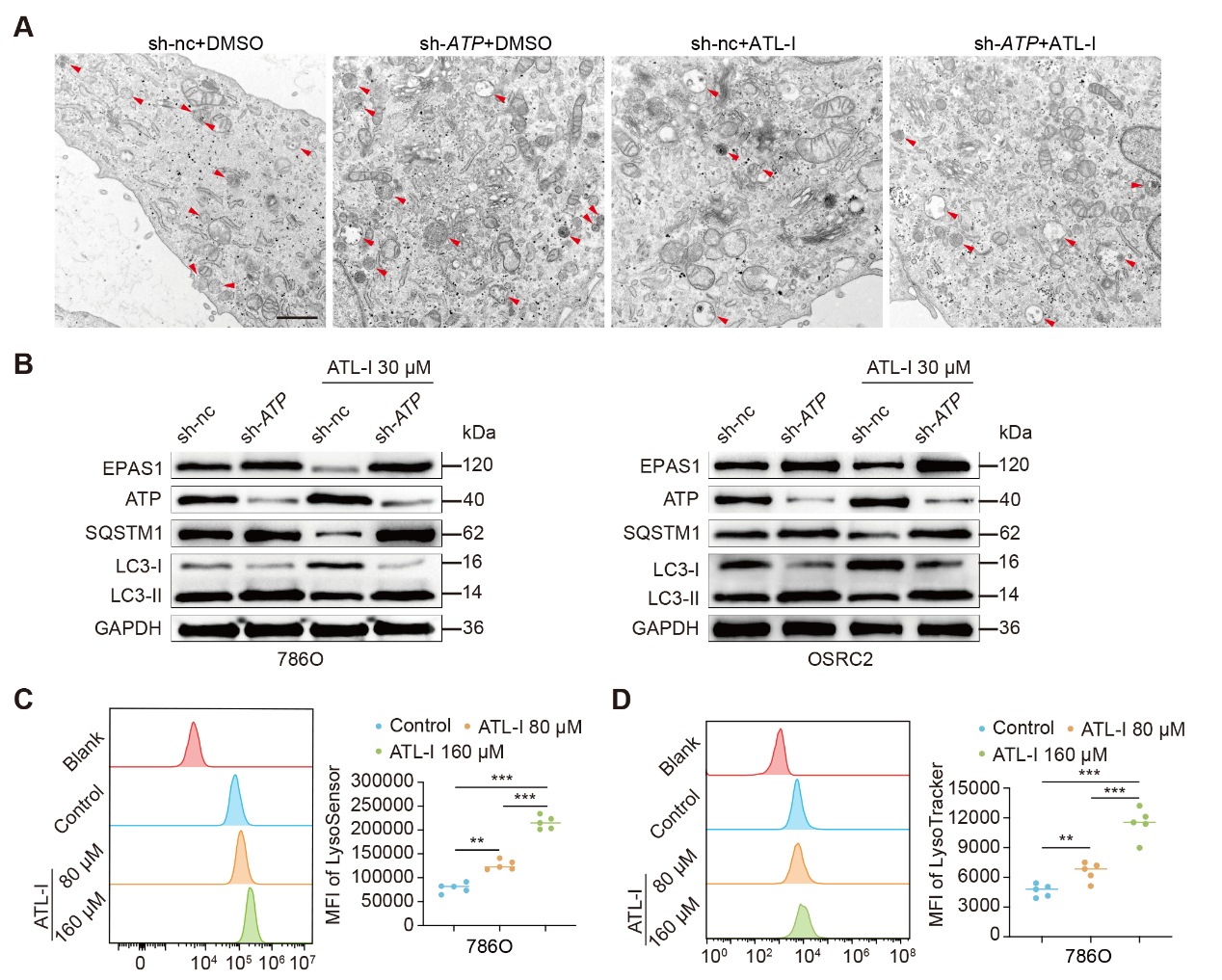
Figure S6.** ATL-I facilitates the fusion of autophagosome and lysosome membranes and enhances lysosomal activity by modulating ATP6V0D2. (**A**) Autophagosome analysis via transmission electron microscopy in sh-nc and sh-*ATP6V0D2* 786O cells or cells treated with 80 µM ATL-I. Scale bar: 1 μm. (**B**) Immunoblotting was conducted to assess the levels of EPAS1, ATP6V0D2, SQSTM1, and LC3-II in 786O and OSRC2 cells with sh-nc and sh-*ATP6V0D2* knockdown, both untreated and treated with 30 μM ATL-I. (**C and D**) Lysosomal acidification and activity were evaluated by flow cytometry with LysoSensor (**C**) or LysoTracker (**D**) staining in 786O cells treated with 0, 80, or 160 µM ATL-I for 48 h. (** p < 0.01, *** p < 0.001.)

**
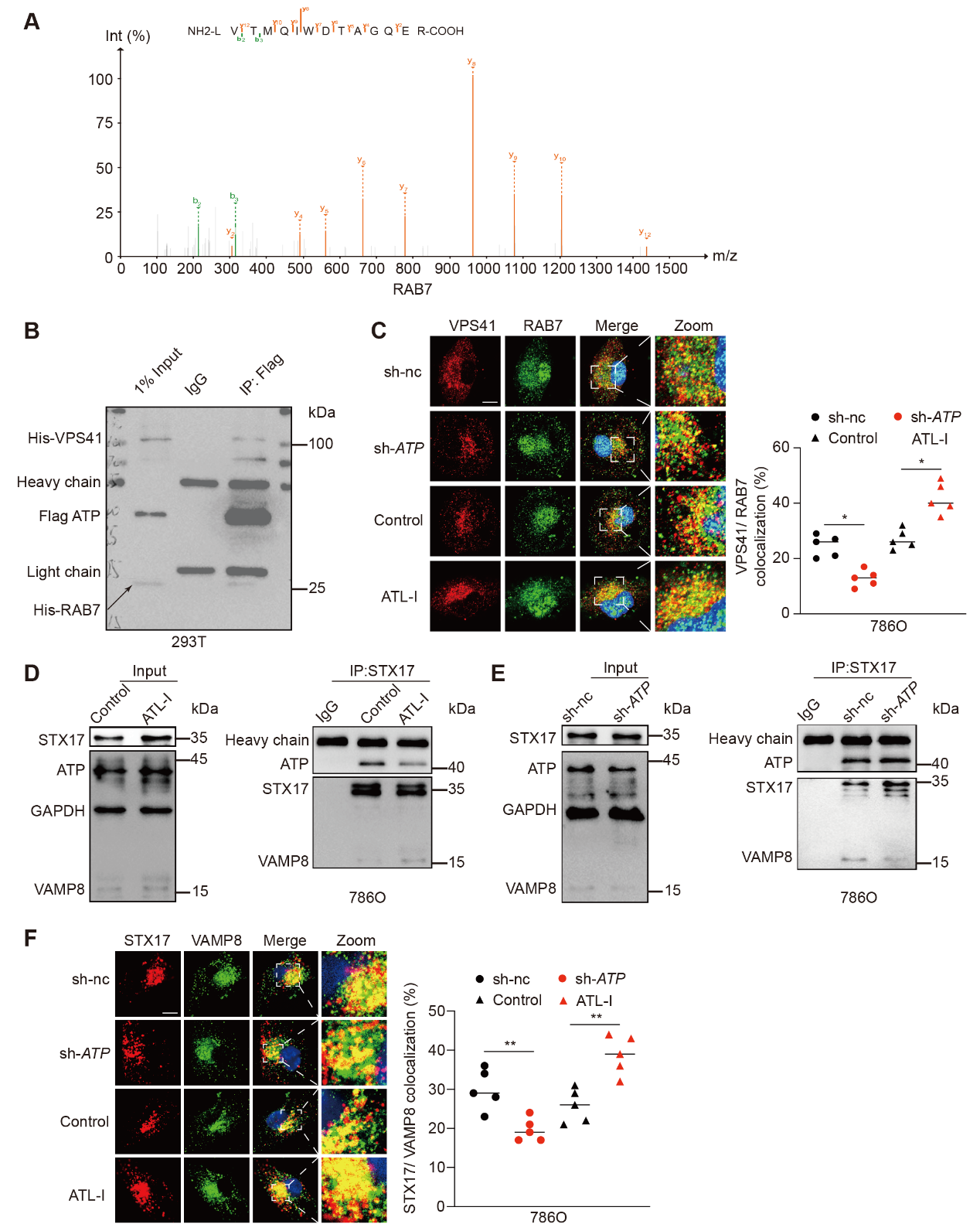
Figure S7.** ATL-I increased the interaction between STX17 and VAMP8 through ATP6V0D2. (**A**) The secondary mass spectrum of RAB7. (**B**) 293T cells stably expressing Flag-ATP6V0D2 were immunoprecipitated by beads. Samples were immunoblotted for exogenous VPS41 and RAB7. (**C**) The indicated cells were stained with anti-VPS41 and anti-RAB7 antibodies. The colocalization of VPS41 and RAB7 was quantified. Scale bar: 10 μm. (**D**) Cells were untreated or treated with 80 μM ATL-I for 48 h and immunoprecipitated with anti-STX17 antibody. The samples were subjected to western blotting and probed with antibodies as indicated. (**E**) sh-nc and sh-*ATP6V0D2* cells were immunoprecipitated with anti-STX17. The samples were subjected to western blotting and probed with antibodies as indicated. (**F**) The indicated cells were stained with anti-STX17 and anti-VAMP8 antibodies. The colocalization of STX17 with VAMP8 was quantified. Scale bar: 10 μm. (* p < 0.05, ** p < 0.01.)

**
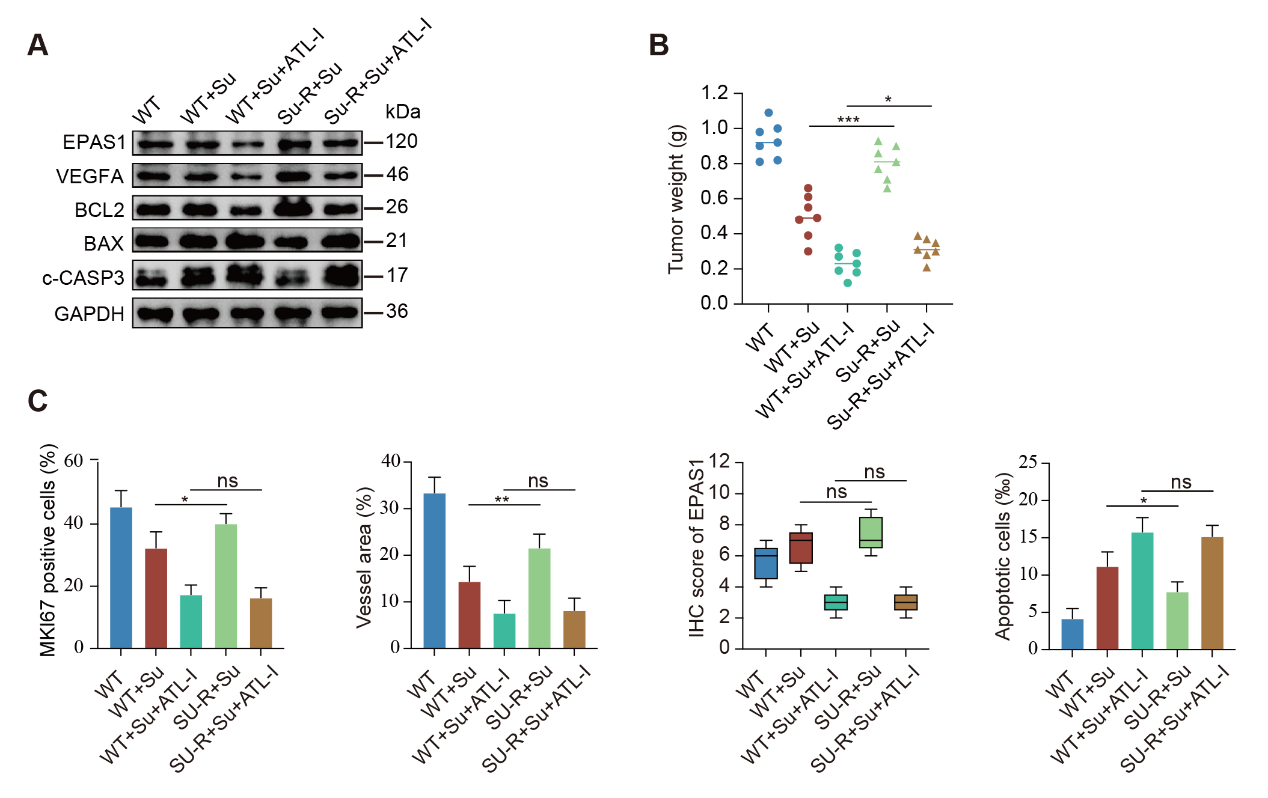
Figure S8.** ATL-I elevated sunitinib responsiveness in ccRCC by inhibiting the EPAS1 pathway. (**A**) Immunoblot analysis of EPAS1, VEGFA, and apoptosis-related markers in the indicated cells treated with 5 μM sunitinib alone or in combination with 80 μM ATL-I for 36 h. (**B**) The tumor weights in the five indicated groups. (**C**) Quantitative analysis of MKI67, PECAM1, and EPAS1 expression and the cell apoptosis ratio in the indicated five groups. (* p < 0.05, ** p < 0.01, *** p < 0.001.)

**Supplementary Tables**

| **Table S1.** Sequence of primers for qRT-PCR analysis. | | |
| --- | --- | --- |
| Primers used in q-PCR analysis | | |
| *GAPDH* | Forward primer | CGCTCTCTGCTCCTCCTGTTC |
|  | Reverse primer | ATCCGTTGACTCCGACCTTCAC |
| *ATP6V0D2* | Forward primer | GAACGTCGAAAAGAAAAGTCTCG |
|  | Reverse primer | AGAGCAAACAAGTGTCGGTCAA |
| *HIF1A* | Forward primer | TCTGTGGACCTGTCGGTGATGG |
|  | Reverse primer | CCTTATCAAGATGCGAACTCACA |
| *EPAS1* | Forward primer | CGGAGGTGTTCTATGAGCTGG |
|  | Reverse primer | AGCTTGTGTGTTCGCAGGAA |
| *VEGFA* | Forward primer | GAGGGCAGAATCATCACGAAG |
|  | Reverse primer | TGTGCTGTAGGAAGCTCATCTCTC |
| *FLT1/VEGFR1* | Forward primer | TGGCCATCACTAAGGAGCACTCC |
|  | Reverse primer | GGAACTGCTGATGGCCACTGTG |
| *KDR/VEGFR2* | Forward primer | CGGACAGTGGTATGGTTCTTGC |
|  | Reverse primer | GTGGTGTCTGTGTCATCGGAGTG |
| *FLT4/VEGFR3* | Forward primer | GTACATGCCAACGACACAGG |
|  | Reverse primer | TGATGAATGGCTGCTCAAAG |
